# High-Throughput Molecular Dynamics-Based Alchemical Free Energy Calculations for Predicting the Binding Free Energy Change Associated with the Selected Omicron Mutations in the Spike Receptor-Binding Domain of SARS-CoV-2

**DOI:** 10.3390/biomedicines10112779

**Published:** 2022-11-01

**Authors:** Rajendra Bhadane, Outi M. H. Salo-Ahen

**Affiliations:** 1 Structural Bioinformatics Laboratory, Faculty of Science and Engineering, Biochemistry, Åbo Akademi University, FI-20520 Turku, Finland; 2 Pharmaceutical Sciences Laboratory, Faculty of Science and Engineering, Pharmacy, Åbo Akademi University, FI-20520 Turku, Finland

**Keywords:** molecular dynamics simulation, SARS-CoV-2, alchemical free energy calculation, binding free energy, spike protein, receptor-binding domain, mutation, omicron

## Abstract

The ongoing pandemic caused by SARS-CoV-2 has gone through various phases. Since the initial outbreak, the virus has mutated several times, with some lineages showing even stronger infectivity and faster spread than the original virus. Among all the variants, omicron is currently classified as a variant of concern (VOC) by the World Health Organization, as the previously circulating variants have been replaced by it. In this work, we have focused on the mutations observed in omicron sub lineages BA.1, BA.2, BA.4 and BA.5, particularly at the receptor-binding domain (RBD) of the spike protein that is responsible for the interactions with the host ACE2 receptor and binding of antibodies. Studying such mutations is particularly important for understanding the viral infectivity, spread of the disease and for tracking the escape routes of this virus from antibodies. Molecular dynamics (MD) based alchemical free energy calculations have been shown to be very accurate in predicting the free energy change, due to a mutation that could have a deleterious or a stabilizing effect on either the protein itself or its binding affinity to another protein. Here, we investigated the significance of five spike RBD mutations on the stability of the spike protein binding to ACE2 by free energy calculations using high throughput MD simulations. For comparison, we also used conventional MD simulations combined with a Molecular Mechanics-Generalized Born Surface Area (MM-GBSA) based approach, and compared our results with the available experimental data. Overall, the alchemical free energy calculations performed far better than the MM-GBSA approach in predicting the individual impact of the mutations. When considering the experimental variation, the alchemical free energy method was able to produce a relatively accurate prediction for N501Y, the mutant that has previously been reported to increase the binding affinity to hACE2. On the other hand, the other individual mutations seem not to have a significant effect on the spike RBD binding affinity towards hACE2.

## 1. Introduction

In the current pandemic caused by SARS-CoV-2 [1], the study of viral mutations can give us understanding on the infectivity, pathogenesis and drug resistance of the virus as well as insight into vaccination efficiency. Further, it may help tracking immune escape routes and consequent emergence of new diseases. During the second wave of COVID-19, SARS-CoV-2 mutations drew much more attention than those of any other virus studied to date. However, it is important to point out that viral mutation is not an unusual phenomenon. For any organism, the mutation rate is the change in genetic information that passes from one generation to the next. The viral mutation rate involves a process that starts from the viral particle’s attachment to the host, its entry, release of genetic material, replication of viral particles, assembly and release or its escape from the host cell [2]. Viruses show a higher mutation rate than other organisms due to their short generation time and large population size. The mutation rate of viruses also depends on the type of virus. RNA viruses show higher mutation rates than DNA viruses due to their error-prone replication [3]. It has also been shown that the greater the speed of replication, the more errors occur [4,5]. For example, the HIV virus shows an extremely high mutation rate due to the high error rate of the reverse transcriptase and a very fast replication process [6]. On the other hand, despite SARS-CoV-2 virus being an RNA virus, mutations of SARS-CoV-2 are slower than those of HIV due to the unique proofreading function characteristic of some coronaviruses [7]. There are also many other factors that could influence the mutation rate of a particular virus. It is also important to understand that not every mutation is beneficial to a virus, as most mutations are deleterious, leading to the instability of the viral particles [6]. The highly deleterious mutation is rapidly purged, while neutral or mildly deleterious mutations remain. These mild mutations impact the virus phenotype and could be beneficial for pathogenicity, infectivity, transmissibility and/or antigenicity of virus particles [8,9].

Since the first outbreak of SARS-CoV-2 in Wuhan, China, the virus has mutated to various lineages. Among these so-called Pango (Phylogenetic Assignment of Named Global Outbreak) [10] lineages, some are classified as variants of concern (VOC), variants of interest (VOI) or variants under monitoring (VUM). According to the simplified nomenclature by the World Health Organization (WHO), Pango lineages such as B.1.1.7, B.1.351, P.1 and B.1.617.2 are named with the letters of the Greek alphabet: alpha, beta, gamma and delta, respectively [11]. Currently (August 2022), the B.1.1.529 lineage (omicron) and its descendants are the dominant variants worldwide. Among the known variants, the omicron sub lineages BA.1, BA.2, BA.3, BA.4 and BA.5 are VOC as of August 2022 (https://www.who.int/en/activities/tracking-SARS-CoV-2-variants/ accessed on 1 August 2022) (BA.2, BA.4 and BA.5 in the EU/EEA countries; https://www.ecdc.europa.eu/en/covid-19/variants-concern, accessed on 1 August 2022). Of special interest in the present work are the mutations occurring in the receptor-binding domain (RBD) of the SARS-CoV-2 spike protein [12,13], since that domain plays a major role in the entry of the virus into the host cell by binding to the human angiotensin-converting enzyme 2 (hACE2). For example, a sub lineage of the alpha variant, which was first detected in the U.K. in December 2020, carried the E484K and N501Y mutations in the spike RBD, and was 30–50% more infectious than the original virus [12]. Amazingly, the very rapidly spreading omicron variant, first spotted in South Africa and Botswana in November 2021, carries more than 50 mutations compared to the original virus, of which more than half contribute to the changes in the spike protein [14].

**Figure 1:**
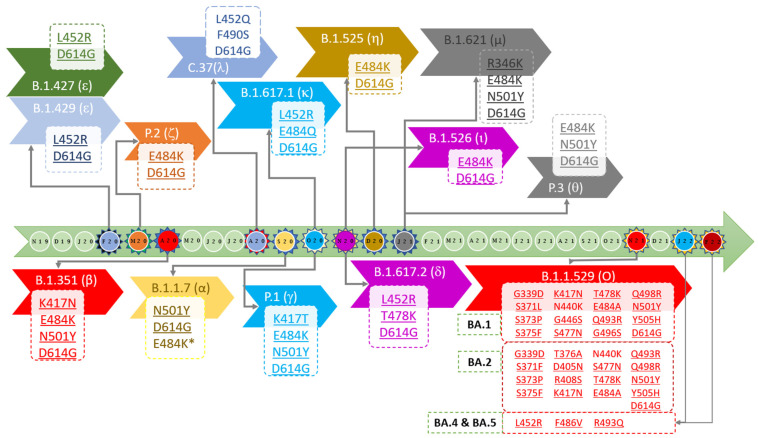
Timeline of various SARS-CoV-2 mutations observed across the Pango lineages. The WHO-proposed naming scheme is shown in brackets. The light green central arrow indicates the progress bar, and each circle represents a month, starting from November 2019 to February 2022. The circles with spike-like projections represent the month of the first appearance of new important lineages. Each chevron arrow is for individual lineages; the rectangle boxes contain the information about the key spike RBD mutations (* only in the B.1.1.7 sub lineage). Information on the previous and current WHO variants of concern are represented under the central progress bar, whereas previous variants of interest or under monitoring are above the bar.

As per the Pango lineage definition, to date, there have been more than 1855 lineages found for SARS-CoV-2 [15]. The B.1 lineages with the D614G mutation in the spike protein observed in early 2020 gave rise to an increase of more than 20% in transmissibility compared to the original strain of SARS-CoV-2 found in Wuhan, China. Other variants also appeared, including many lineages with E484K as the common mutation, which was found to reduce the binding affinity of antibodies [16,17,18,19,20]. Figure 1 shows the rough timeline of VOC, VOI and VUM lineages with prominent mutations in the RBD region of the spike protein.

The epsilon variant (ε; B.1.427 and B.1.429), with a L452R mutation in the spike RBD, surfaced in the California region of the USA in March 2020. The variant showed a 20% increase in transmission [21], a decrease in susceptibility towards the monoclonal antibody combination containing bamlanivimab and etesevimab and reduced neutralization by convalescent and post-vaccination sera [21,22,23]. The P2 variant (zeta, ζ) originated in Brazil in April 2020 and, similarly to epsilon, also showed reduced neutralization by convalescent and post-vaccination sera and monoclonal antibody treatments. At about the same time, South Africa saw a significant peak in COVID-19 infections, which was confirmed to be caused by the emergence of the beta variant that carries the K417N, E484K and N501Y mutations in the spike RBD. Both the K417N and N501Y mutations have been shown to increase the binding affinity of SARS-CoV-2 RBD to hACE2 [24]. It has been estimated that the beta variant had a 50% increase in transmission rate, and a significantly reduced susceptibility towards bamlanivimab and etesevimab monoclonal antibody treatment [25,26,27]. The gamma variant originating from Brazil also had an increased transmissibility and resembled the beta variant, but had a K417T mutation instead of K417N. In spring 2021, India saw a significant peak in the number of SARS-CoV-2 infections due to the delta variant (B.1.617.2) [28], which had a significantly increased infectivity and viral spread. This variant carries the L452R and T478K mutations at RBD. The other variants also carry different combinations of the aforementioned mutations. The lambda (λ) variant is classified under C.37 lineage, and was reported for the first time in Peru in August 2020. This variant, with L452Q and F490S mutations at RBD, had also improved transmissibility and ability to evade vaccine elicited antibodies [29,30], while the following variant (B.1.1.7, alpha) was known for about a 50% higher transmission rate than the original SARS-CoV-2 virus [12]. Due to the transmission advantage of the alpha variant and its sub lineage (B1.1.7 + E484K) over B.1 and B.1.1 lineages, it became the dominant variant in the UK in the end of 2020, spreading to other parts of the world. The kappa variant (B.1.617.1), first detected in India, carries the L452R and E484Q mutations and has been shown to have an increased transmutability [31]. The mu variant (B.1.621) from Columbia carries an additional R346K mutation not observed in the other variants, and it has shown significant resistance to antibodies elicited by both vaccination and natural SARS-CoV-2 infection [32]. The iota and eta variants, with E484K mutation at RBD, were detected in November and December 2020 in the USA and Nigeria, respectively. In January 2021, the theta variant was reported in the Philippines. It carries E484K and N501Y mutations. The latest VOC, omicron, carries fifteen mutations at RBD [33], of which some are the same as those detected in the previous variants: K417N, T478K and N501Y (+E484A instead of E484K/Q).

Several research groups have experimentally studied the impact of various SARS-CoV-2 mutations in order to shed light on the viral infectivity, spread of the disease or on how the virus escapes from antibodies [34,35,36,37,38,39,40,41,42]. In addition to the time-consuming and costly mutational studies, there are also various computational approaches to predict the effect of single mutations on protein–protein binding affinities [43,44,45,46,47,48,49]. Of the computational approaches, especially molecular dynamics (MD), simulations-based free-energy calculations have been shown to produce useful (relative or absolute) predictions on free-energy changes upon single mutations [50,51,52,53,54,55,56,57]. The exponential growth in computational power has facilitated the utilization of such computationally expensive calculations. Examples of these methods include the ensemble-based Molecular Mechanics-Generalized Born Surface Area (MM-GBSA) and Molecular Mechanics-Poisson–Boltzmann Surface Area (MM-PBSA) [58] approaches, as well as so-called alchemical free energy simulations. In the MM-GB/PBSA method, an implicit solvent model is used for reanalyzing the ensemble of complex conformations in order to avoid the large energy fluctuations due to the explicit solvent molecules that were used in the MD simulation. In an “*alchemical*” transformation, an amino acid is transformed from one state to another via a non-physical pathway. This transformation can be either reversible or irreversible, and can be performed by an equilibrium (e.g., Free Energy Perturbation [59] or Thermodynamic Integration [60]) or non-equilibrium method (e.g., Bennett’s Acceptance Ratio [61] or Crooks’ Gaussian Intersection [62]), respectively [63]. The major advantage of the alchemical approach is the prediction accuracy that matches that which was reached in experiments [64,65].

In this work, we computationally investigated the impact of selected individual mutations of SARS-CoV-2 spike RBD residues on the RBD affinity towards hACE2. We performed alchemical free energy calculations, utilizing high throughput molecular dynamics (MD) simulations to predict the binding free energy change (with respect to binding partner ACE2) upon five spike RBD mutations observed in the omicron variant. We performed, altogether, 20 equilibrium and 2000 non-equilibrium MD simulations in order to ensure exhaustive conformational sampling of each of the mutants. We also carried out comparative studies with conventional MD simulations coupled to the MM-GBSA approach, and discussed the results in light of the available experimental data. The structural insights obtained in this study may allow for further understanding of the impact of these mutations on the infectivity and spread of SARS-CoV-2. Most importantly, we show that the relatively fast free energy calculations may provide a reasonable alternative for expensive experimental mutation studies.

## 2. Results and Discussion

### 2.1. Selection of Mutations for the Study

Based on the reports on increased infectivity of alpha and beta variants and the sudden spike in infections due to omicron variant, the key mutations in the spike RBD of these variants were selected for the study. In omicron variants, the following mutations are present in the RBD region: G339D, S371L, S373P, S375F, K417N, N440K, G446S, S477N, T478K, E484A, Q493R, G469S, Q498R, N501Y and Y505H. As alchemical calculations are generally very resource-intensive, requiring several hundred MD simulations per mutation, we selected only five non-charge changing mutations from the key RBD mutations for this study: G446S, F486V, G496S, N501Y and Y505H. We chose to study their individual impact on the spike RBD-hACE2 affinity rather than their combined effect. To our knowledge, there are no virus variants that would contain only these mutations, which could justify the study of their realistic combined effect. On the other hand, experimental studies have explored the individual effects of these mutations, which makes it possible to compare our computational results with those data (see Table 1). For two mutants, the experimental spike-hACE2 binding affinity (K_d_) has also been reported. All of the mutants have been shown to evade antibodies, at least to some extent.

### 2.2. Location of the Selected Mutations at the Receptor-Binding Domain of the Spike Glycoprotein

**Figure 2:**
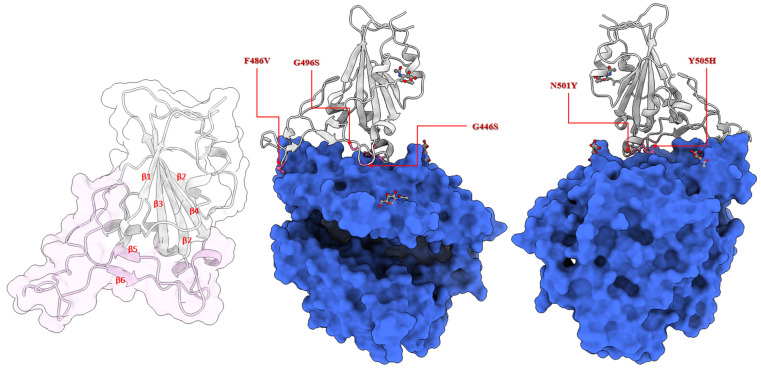
Location of the studied mutations in the spike glycoprotein receptor-binding domain (RBD) of SARS-CoV-2 (PDB ID: 6LZG). From left to right: transparent surface and cartoon representation of the spike RBD (colour scheme: the receptor-binding motif (RBM) domain in pink, the beta sheets are labeled); spike RBD in complex with hACE2 front view and back view (colour scheme: spike RBD in grey cartoon, hACE2 in blue surface, locations of labeled mutations in pink ball and stick representation, spike RBD and hACE2 glycosylation sites in elemental ball and stick representation.

The envelope of SARS-CoV-2 has homotrimeric spike glycoproteins that consist of a S1, S2 and a short cytoplasmic domain. In order to interact with the human angiotensin-converting enzyme 2 (hACE2) the virus utilizes the amino acids 331–524 of the S1 domain, that is, the receptor-binding domain (RBD) [79]. In order to structurally analyze the selected mutations, we searched for the available 3D structures of the spike RBD bound with the hACE2 receptor. Currently, there are hundreds of SARS-CoV-2 spike protein structures available in the Protein Data Bank (PDB), of which tens of structures are bound with hACE2. These include structures determined using both X-ray crystallography and cryo-EM. The resolution of these structures varies between 2 and 5 Å. We set the resolution criteria of 2.0–3.0 Å. As we focused on the RBD of the spike protein, we ignored the structures of full-length spike glycoproteins. We chose the structure deposited by Wang et al. (PDB ID: 6LZG, resolution 2.5 Å) [79] for this study. The characteristic feature of the spike RBD core region (residues 333–437, 507–527) is a twisted, five-stranded β-sheet (formed by antiparallel β1–4 and β7 strands) with short connecting helices (α1-α3) and loops. Between β4 and β7 strands, there is an external subdomain (residues 438–506) that is formed by α4 and α5 helices and a flexible loop which connects two short β strands (β5 and β6). This subdomain is also called the receptor-binding motif (RBM) region, since it makes direct contact with the hACE2 receptor. All selected mutations are in the RBM region. G446S and G496S are in the flexible loop region close to the β5 and β6 strands, respectively. F486V lies also in the flexible loop region before the β6 strand. The Y505H mutation is located close to the β7 strand, while N501Y precedes the α5 helix [80]. Figure 2 shows the location of the mutations selected to be studied here, as well as the RBD and hACE2 glycosylation sites. The glycosylation sites are not in direct proximity to the studied mutation sites, which suggests that the glycans at these sites would not, at least directly, interfere with the effects of these mutations.

### 2.3. Conventional MD Simulation Studies

The structures of the wild type (WT) and mutant spike RBD bound to hACE2 were used as input for conventional MD simulations. For each selected mutation, three replicate simulations of 200 ns were performed. The Y505Hε (histidine protonated at the epsilon ε nitrogen) mutant was simulated for 300 ns. Averaged and individual simulations’ root-mean-square deviation (RMSD) and root-mean-square fluctuation (RMSF) plots are shown in Figures S1–S4, as well as pages S2–S4 and S6–S8 in Supporting Information. The averaged RMSD of the G496S and Y505Hδ (histidine protonated at the delta δ nitrogen) mutants and the WT spike RBD converges early at 40 ns, followed by that of G446S and F486V at 60 ns, and remains stable throughout the MD simulations. The averaged RMSD of the N501Y mutant converges only after 90 ns, and remains stable until the end of the simulation. This is due to the second replica simulation, which shows a large RMSD jump at around that time point, apparently due to floppiness of the flexible loop region (residues 481–484) (Supporting Information, page S8). Small average RMSD fluctuations of less than 0.5 Å were observed for F486V and G446S during the simulations. The RMSF values for the loop (residues 481–484) preceding residue 486 were generally varying significantly (between 1.5 Å and ca. 4 Å), depending on the replica simulation. For the F486V mutant, two replica simulations of the three showed the highest RMSF peak for this loop, with the top value being 4.4 Å (Supporting Information, page S7, Figure S4). Somewhat greater RMSD fluctuations of about 1–2 Å can be seen for the Y505Hε (histidine protonated at the epsilon ε nitrogen) mutant at about 85–100 ns and 190–230 ns, respectively, and the RMSD stabilizes only at about 235 ns. This is caused by the large fluctuations in the first replica simulation (Supporting Information, Figure S2). The fluctuations are prominent at the C-terminal end of the Y505Hε mutant (Supporting Information, Figure S3, page S8).

**Figure 3:**
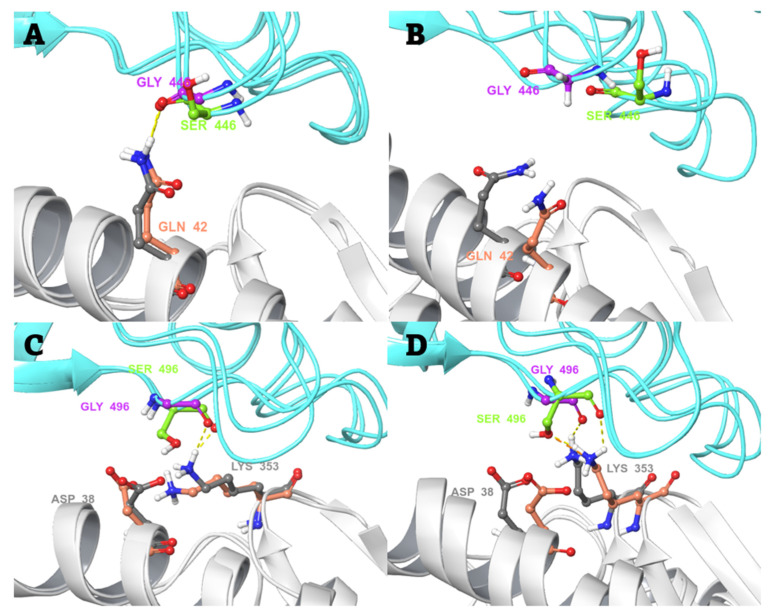
The change in local environment at the binding interface of SARS-CoV-2 spike RBD and hACE2 is caused by single-residue mutations. The orientation and interactions of Gly446 of WT spike RBD and Ser446 of the mutant, with respect to Gln42 of hACE2 (**A**) before the start of production MD simulation, and (**B**) after a 200-ns MD simulation. The orientation and interactions of Gly496 of spike RBD WT and Ser496 of the mutant, with respect to Asp38 and Lys353 of hACE2 (**C**) before the start of production MD simulation, and (**D**) after a 200-ns MD simulation. Colour scheme: spike RBD and hACE2 in cyan and white colour cartoon, respectively. Spike RBD and hACE2 amino acid residues in pink/grey (WT complex) and green/salmon (mutant complex) elemental ball and sticks representation, respectively. Interactions are shown in dashed lines: yellow—H-bonds; orange—bad contacts.

In case of the G446S mutant, there is no significant difference between the interactions of the WT Gly446 or the mutant Ser446 with Gln42 of hACE2, as can be seen from the snapshot structures taken before and after simulations in Figure 3A,B. During the simulation, both residues tend to lose the initial hydrogen bond between the backbone oxygen of the RBD residue and the Gln42 sidechain nitrogen of hACE2. Moreover, the main chain phi and psi angles of Ser446 move from the additionally allowed region in the Ramachandran plot to the most favorable region during the simulation (while Gly446 remains in the additionally allowed region), which likely explains the slight shift in the position of Ser446. On the other hand, in the case of the G496S mutant, the change from glycine to serine retains the hydrogen bond interaction between Lys353 of hACE2 and the backbone oxygen of the spike RBD residue. However, there is a slight steric clash between the lysine side chain nitrogen and the serine side chain hydroxyl group in the mutant (Figure 3C,D).

**Figure 4:**
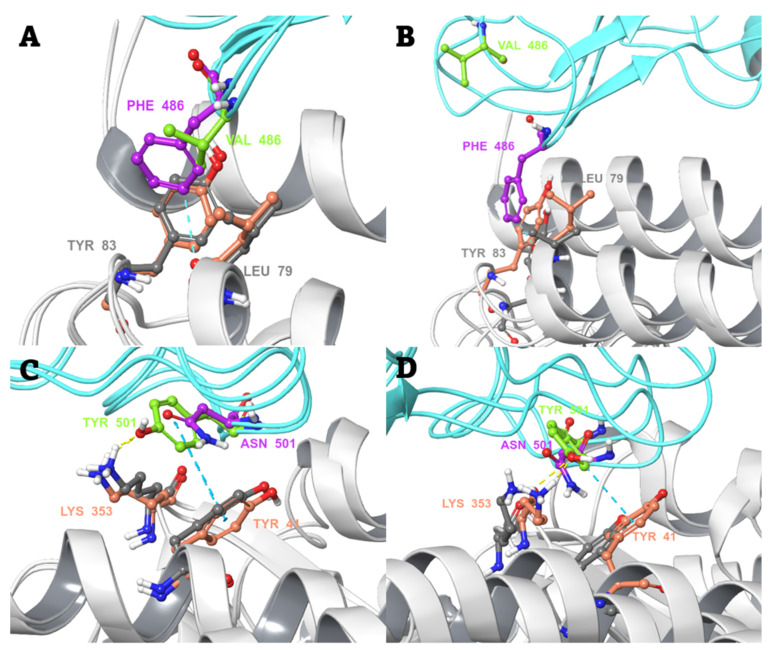
The change in local environment at the binding interface of SARS-CoV-2 spike RBD and hACE2 caused by single-residue mutations. The orientation and interactions of Phe486 of WT spike RBD and Val486 of the mutant, with respect to Leu79 and Tyr83 of hACE2 (**A**) before the start of production MD simulation, and (**B**) after a 200-ns MD simulation. The orientation and interactions of Asn501 of spike RBD WT and Tyr501 of the mutant, with respect to Tyr41 and Lys353 of hACE2 (**C**) before the start of production MD simulation, and (**D**) after a 200-ns MD simulation. Colour scheme: spike RBD and hACE2 in cyan and white colour cartoon, respectively. Spike RBD and hACE2 amino acid residues in pink/grey (WT complex) and green/salmon (mutant complex) elemental ball and sticks representation, respectively. Interactions are shown in dashed lines: yellow—H-bonds; light cyan—aromatic H-bonds; cyan—pi-pi interactions.

In the case of the F486V mutant (Figure 4A,B), the mutation of the aromatic bulky phenylalanine to the smaller valine removes the pi-pi interactions (Supporting Information, Figure S4) and diminishes the van der Waals interactions with the neighbouring Tyr83 and Leu79 of hACE2, respectively. In addition, the occasional aromatic hydrogen bond between Phe486 and Leu79 backbone oxygen (Figure 4A,B) is lost. This causes an apparent destabilization of the complex, as the mutant RBD loop moves away from the initial interaction site during the MD simulation. This seems to also relate well to the observed greater averaged RMSF values of Val486 and the preceding residues compared to the WT (Supporting Information, Figure S4). The opposite effect was expected from the N501Y mutant, where the polar asparagine is replaced with the aromatic tyrosine. Tyr501 forms optimal interactions with the neighbouring Tyr41 and Lys353 residues of hACE2 (pi-pi stacking and a hydrogen bond, respectively) (Figure 4C,D), thus stabilizing the binding interaction with hACE2. These new interactions prevailed during the MD simulations. For example, the phenyl ring of the mutant Tyr501 stays very close (within 4.2–6.5 Å) to the phenyl ring of Tyr41 of hACE2 and, thus, is engaged in pi-pi stacking interactions throughout the 200-ns MD simulation (although there is some variation between the three replica simulations) (Supporting Information, Figures S6 and S7).

**Figure 5:**
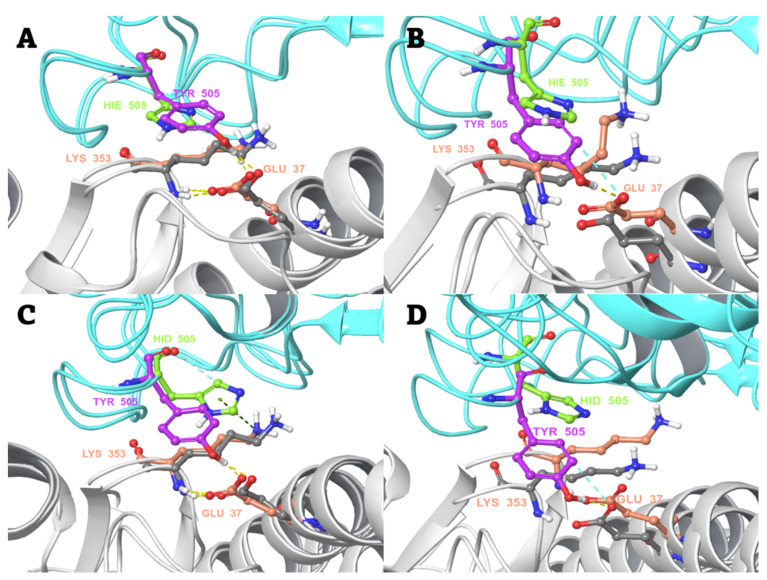
The change in local environment at the binding interface of SARS-CoV-2 spike RBD and hACE2 caused by Y505H mutation. The orientation and interactions of Tyr505 of WT spike RBD and HIE505 (protonated ε nitrogen) of the mutant, with respect to Glu37 of hACE2 (**A**) before the start of production MD simulation, and (**B**) after a 200-ns MD simulation. The orientation and interactions of Tyr505 of spike RBD WT and HID505 (δ protonated nitrogen) of the mutant, with respect to Glu37 of hACE2 (**C**) before the start of production MD simulation, and (**D**) after a 200-ns MD simulation. Colour scheme: spike RBD and hACE2 in cyan and white colour cartoon, respectively. Spike RBD and hACE2 amino acid residues in pink/grey (WT complex) and green/salmon (mutant complex) elemental ball and sticks representation, respectively. Interactions are shown in dashed lines: yellow—H-bonds; light cyan—aromatic H-bonds; green—pi–cation interactions.

In case of the Y505H mutant (Figure 5A–D), independent simulations were performed for the alternative (neutral) protonation states, i.e., either epsilon (ε) or delta (δ) nitrogen protonated). It was observed that in WT spike RBD, Tyr505 maintains the initial strong hydrogen bond interaction between its hydroxyl group and the side chain oxygen of hACE2 Glu37 during the MD simulations. An additional aromatic hydrogen bond between the tyrosine ring and the Glu side chain oxygen is also possible. The tyrosine-to-histidine mutation causes a loss of this important interaction, no matter the histidine protonation state. Hence, this mutation alone may have a deleterious effect on hACE2 binding, despite a possible pi-cation interaction with Lys353 (Figure 5C).

In order to estimate the contribution of the WT and mutated spike RBD residues to the binding affinity towards hACE2, binding free energy calculations were performed by Schrödinger’s Prime/MM-GBSA method. It is a single trajectory method, and thus is appropriate for calculating mainly relative binding free energies of the WT and the mutants binding to the common protein [81]. Snapshots from the MD simulations of the spike RBD-hACE2 complexes were taken, assuming that the conformations of both the ligand (spike RBD) and the receptor (hACE2) are the same in the bound and the free state, and no major conformational changes occur upon binding.

**Figure 6:**
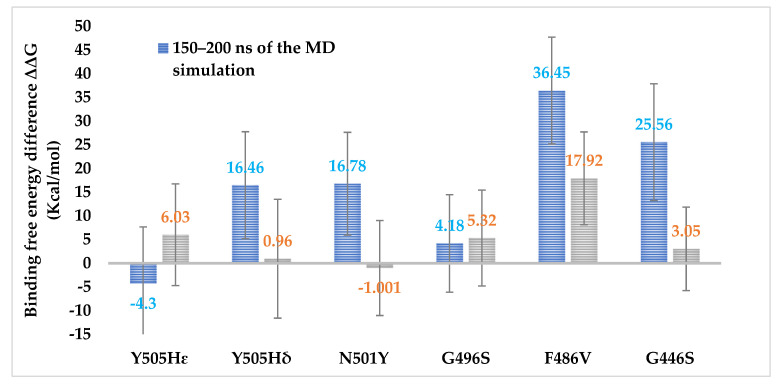
The average change in binding free energy (ΔΔG) between the SARS-CoV-2 WT spike RBD and its mutants in unbound and bound conformation, with hACE2 calculated by the Prime/MM-GBSA method. The calculations were performed using both the first 1–25 ns and last 50 ns of the 200-ns (300 ns for the Y505HIE mutant) MD simulations in three replicas.

Since the conformational changes at the protein–protein binding interfaces can be fairly significant the longer the MD-simulation lasts, we investigated the effect of the length of the simulation on the Prime/MM-GBSA energy values. Figure 6 shows the average change in binding free energy (ΔΔG) upon mutation, based on the trajectory snapshots taken from the beginning of the simulation (1 ns–25 ns) and the end of the simulation (150–200 ns; 250–300 ns for Y505HIE mutant) (average of three replicates).

We observed that the trajectory part used for the MM-GBSA calculations affected the resulting ΔΔG values significantly in almost all mutant cases. For the G496S mutant the difference in the values calculated from the beginning vs. the end of the simulation was the smallest. All mutations but Y505HIE were predicted to decrease (some even drastically) the spike RBD-hACE2 binding affinity when looking at the values calculated from the end of the 200-ns MD simulation. Notably, the absolute Prime/MM-GBSA energy values are usually not directly comparable with the experimental values, and the rescale factor is system dependent [82]. On the other hand, the resulting ΔΔG values from the beginning of the trajectory seem to be more consistent with the structural insights we presented above, N501Y being the only mutant increasing the binding affinity. However, the standard deviations (SD) are large, and only the F486V mutation is predicted to be clearly deleterious with the most positive ΔΔG values (including the SD, the values are all the time above zero). The effect of the simulation time on binding affinity prediction by MM-GBSA and MM-PBSA methods has been briefly discussed in the literature [83,84]. Most of these studies were performed for ligand-receptor complexes with relatively short simulation timescales (1–10, even 40 ns) [83,84,85]. Depending on the system, a longer simulation can be beneficial, but not always. The MM-GBSA calculations performed for the snapshots from the first 1–25 ns of the simulation could be regarded as comparable for such studies with short simulations. Accounting for protein induced fit effects upon ligand binding or achieving convergence in sampling, relevant conformations may speak to the need for longer simulation times [86]. However, longer simulations do not necessarily give more accurate binding free energy values if the force field is not precise, at least when only a single trajectory is used [87]. In the case of the MM-GBSA calculations performed on the trajectory frames from 150–200 ns of the simulation, it is possible that the protein–protein complex already starts to dissociate, at least partially, from the flexible loop areas and edges if there are no strong interactions (see, for example, F486V in Figure 4B). This, of course, affects the binding free energies significantly. There is an apparent paradox between the simulation time needed for achieving equilibrated RMSD values for large protein–protein complexes and the commonly used short time frames for binding free energy calculations, using MM-GBSA to avoid bigger conformational changes at the binding site. This may, of course, be case-dependent, and needs further investigation.

### 2.4. MD-Based Alchemical Free Energy Calculations

Despite running replicate simulations, limited sampling during the conventional MD simulations might affect the ability to predict the stability of the interactions upon mutation. Hence, in order to further increase the conformational sampling of the mutants, we carried out high-throughput MD simulations, utilizing non-equilibrium alchemical free energy calculations to investigate the effects of these mutations on hACE2 binding. In the current work, a two-trajectory method was used: simulations of both WT and mutant RBD were performed in bound and unbound state. Table 2 contains the resulting binding free energies calculated for both the bound and unbound states (Figures S8–S13, Supporting Information) and the change in binding free energies upon mutation (ΔΔG).

According to the non-equilibrium free energy calculations, N501Y is the most significant stabilizing mutation affecting the binding of spike RBD to hACE2. This is followed by G446S, which is predicted to have a mild stabilizing effect. As we know, the N501Y mutant has been experimentally verified to increase the binding affinity (Table 1), whereas the G446S mutation has been shown to slightly (by 1.6-fold) decrease the binding affinity [66,67]. The remaining three F486V, G496S and Y505H mutations are predicted to be mildly to moderately destabilizing. Recently, indeed, the F486V and Y505H mutations have been reported to compromise hACE2 binding affinity [69,70,71], and it has been shown that the G496S mutation causes a two-fold reduction in binding affinity [69]. The predictions for the direction of the affinity change by Prime/MM-GBSA (from the first 1–25 ns of the MD simulation) and the alchemical free energy method are qualitatively consistent for F486V, G496S, N501Y and Y505H, while they differ for the G446S mutant.

**Figure 7:**
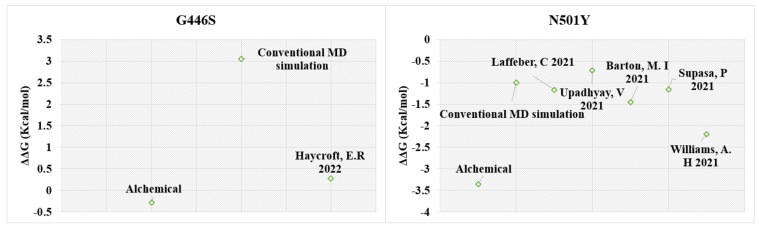
Predicted versus experimentally reported binding affinity of spike receptor-binding domain with hACE2 for the G446S and N501Y mutations . The experimental values were taken from Table 1 and converted to ∆G [40,66,74,77,78]. The ∆∆G was calculated by subtracting the binding free energy of the WT spike (∆G_WT_) from that of the mutant spike (∆G_MUT_). The Prime/MMGBSA method was combined with the conventional MD and carried out for the first 1–25 ns of the simulation trajectory. The alchemical free energy method was carried out with the PMX software using a non-equilibrium method.

We then quantitatively compared the predicted binding affinity of the G446S and N501Y mutants with the experimentally reported binding affinities (Figure 7, Table 1, Supporting Information, Table S3).

For the G446S mutant, the alchemical method predicts a ∆∆G value of −0.28 ± 0.07, whereas the Prime/MM-GBSA method predicts a change in binding free energy of 3.05 ± 8.8 kcal/mol (calculated from the beginning of the simulation). Since the experimentally determined value is 0.28 ± 0.12 kcal/mol (Figure 7, Table S1), we can conclude that the alchemical method is quantitatively more accurate, despite the ∆∆G value being in the opposite direction. For the N501Y mutant, both the Prime/MM-GBSA method combined with the conventional MD-simulations and the alchemical free energy calculations produce ∆∆G values which are comparable to the experimental values (Figure 7). Both computational methods were successful in predicting the positive effect of the N501Y mutation on the hACE2 binding affinity, although the Prime/MM-GBSA values have a very large error, and the results are strongly dependent on the simulation length. While the experimental range of ∆∆G varies from −2.2 to −0.72 ± 0.23 kcal/mol (Figure 7, Table S1), the Prime/MM-GBSA prediction of −1.0 kcal/mol (from 1–25 ns of the simulation) seems to compare well with the highest experimental values, and the prediction of −3.35 ± 0.23 kcal/mol by the alchemical approach is nearer to the lowest experimental value (Table 1 and Table 2). However, the SD for the Prime/MM-GBSA predicted value is very large (±10.01), which indicates that more sampling would be needed. In general, the SD values for the results from the alchemical method are much smaller, as they are for every mutant significantly smaller than the ∆∆G value itself (whereas the opposite is true for most of the Prime/MM-GBSA predictions, i.e., SD being greater than the actual predicted ∆∆G). Notably, for the alchemical method, the analytical error for the BAR estimator is sensitive to the lack of overlap between the forward and reverse work distributions [86]. The lack of overlap may be due to the lack of convergence during the non-equilibrium transitions. There seems to be lack of overlap between some of the work distributions (bound or unbound states) in case of the G446S, G496S and Y505H mutants (Supporting Information, Figures S8–S13). Thus, the predicted values may not be completely accurate, and more sampling may be needed. On the other hand, very small changes in binding free energies may be difficult to calculate reliably, due to the accuracy limits of non-polarizable force fields used here, such as OPLS4 and Amber99SB. It is also challenging to interpret the biological significance of such small effects.

## 3. Conclusions

The viral mutation is an ongoing process and can evolve in response to selective pressures. The SARS-CoV-2 mutation pattern on spike proteins observed to date suggests that during the first phase of COVID-19 infections, mutations with increased infectivity overcame the early variant. This can be seen for example from the disappearance of B.1 and B.1.1 lineages and their replacement by more infectious B.1.1.7 lineage in the UK. Though both B.1 and B.1.1.7 carry the characteristic D614G mutation that is known to cause a 20% increase in transmission efficiency, the latter, in addition to D614G, has the N501Y mutation that ensures more efficient binding of spike RBD to hACE2 [12]. The N501Y mutation evolved as one of the most efficient mutations, which has continued in all omicron variants.

Consistent with our computational results, previously reported investigations have shown that N501Y stabilizes the interaction between the spike protein and the hACE2 receptor, thus increasing infectivity [21,88,89,90]. The slight destabilizing effect predicted by the alchemical method for the F486V, G496S and Y505H mutations is consistent with the recently published experimental results. On the other hand, the prediction of the effect of G446S on the spike RBD binding affinity to hACE2 is not completely consistent with the experimental data, which most likely depends on the prediction accuracy and, thus, convergence of sampling.

For an ideal free-energy prediction method, the predictive accuracy should be in the same range as that obtained by experimental methods. When both computational approaches were compared, we observed a great difference in the accuracy of the methods. There is an apparent qualitative consensus between the Prime/MM-GBSA and the alchemical method, if we use the beginning of the MD trajectory for the MM-GBSA calculations and ignore the large SD values. However, using the end of the longer simulation for the MM-GBSA calculations gives different results. This suggests that for studying the effect of a mutation on binding affinity, an extended simulation would not give more accurate results (although the relative error, i.e., the SD value with respect to the ∆∆G, decreases in the case of some of the mutants). However, shorter simulations may also not suffice without proper convergence of sampling.

Quantitative comparison of the methods is also not possible, due to the system-dependent scaling factor of the Prime/MM-GBSA method. We also need to remember the approximations in the single trajectory method, which make the method more suitable for predicting relative binding free energies than absolute energies [91]. Differences in binding free energy values may also be partly due to the different force fields that are employed in these methods. However, the OPLS forcefield (in Prime/MM-GBSA) has been significantly improved during the recent years; for example, the previous OPLS3 force field version performed comparably in protein simulations when compared with the state-of-the-art Amber and CHARMM force fields [92]. Of course, some differences are still expected when calculating binding free energies. Nonetheless, the root-mean-square error in kcal/mol of relative binding free energies for a wide range of ligand–protein complexes was shown to be less than 1 kcal/mol.

Despite the quantitative and/or qualitative inaccuracies in predicting the impact of the individual mutations, conventional MD simulations could shed light on the binding interactions at each mutation site. These insights were also indicative of the possible impact on the binding free energies.

In sum, the results from the alchemical method are consistent with the available experimental data on all but one (G446S) of the selected mutations. Quantitatively, however, that prediction is much closer to the experimental value than the MM-GBSA predicted value. Overall, the alchemical method outperforms the less rigorous MM-GBSA method and could accurately predict the stabilizing effect of the N501Y mutation on the binding interactions between spike RBD and hACE2.

## 4. Materials and Methods

### 4.1. Protein Preparation

The 3D structure of the spike receptor binding domain (RBD) that is complexed with the angiotensin-converting enzyme-2 (ACE2) (PDB ID: 6LZG [79]) was retrieved from the Protein Data Bank (PDB) [93]. The selected structures were processed using the Protein Preparation Wizard [94] of Maestro (Schrödinger Release 2021-2 New York, NY, USA). Briefly, the missing hydrogen atoms were added to the structure, and the hydrogen bond network was optimized using PROPKA at pH 7.0. All water molecules beyond 3 Å were removed, and a restrained minimization was conducted using the OPLS4 [95] force field with a convergence criterion of 0.3-Å root-mean-square deviation (RMSD) for all of the heavy atoms.

### 4.2. Conventional MD Simulations

The WT and mutated structures of spike RBD in complex with hACE2 were submitted to a 200-ns molecular dynamics (MD) simulation in three replicates, using the Desmond MD engine as implemented in Maestro (Schrödinger Release 2021-2: Desmond Molecular Dynamics System, D. E. Shaw Research, New York, NY, USA, 2021) [96]. The Desmond simulations were performed with the OPLS4 [95] force field. The simulation systems were prepared using the System Builder tool of the Desmond module. The single point charge (SPC) water [97] was chosen as the explicit solvation model. Each system was neutralized using an appropriate number of Na^+^ or Cl^−^ counter ions. An orthorhombic simulation box, with Periodic Boundary Conditions (PBC) and a 10-Å buffer space between the solute and the box edge, was used for each system. The simulation systems were minimized and equilibrated before the production simulation in a stepwise manner. After the system relaxation, the production simulation was performed in the NPT ensemble for 200 ns (300 ns for the Y505HIE mutant), using a reversible reference system propagation algorithm (RESPA) integrator [96]. The temperature (300 K) was set using the Nosé–Hoover chain thermostat [98,99,100], with a relaxation time of 1.0 ps. The pressure was set at 1.01325 bar with the Martyna–Tobias–Klein barostat [101], using isotropic coupling and a relaxation time of 2.0 ps. Long-range interactions were handled using the U-series method [102], and for short-range interactions, a cut-off radius of 9.0 Å was used. The MD trajectories were analyzed using the Maestro built-in Simulation Interactions Diagram and simulation event analysis tool, and raw data obtained from simulations trajectories was used to plot graphs in Microsoft Excel 365.

### 4.3. Prime/MM-GBSA Binding Free Energy Analysis

The binding free energy between the spike RBD (WT or mutants) and hACE2 was calculated from 25 snapshots of the first 1–25 ns and 100 snapshots of the last 50 ns of the conventional MD simulation trajectory (as three replicas) using the Prime/MM-GBSA approach, as implemented in Schrödinger’s Maestro [103,104] with an implicit solvation model VSGB2.1 [105] and OPLS4 force field. Prime/MM-GBSA method works by calculating the ΔG_bind_ using the following expression:

∆G_bind_ = G_hACE2-spikeRBD_ − (G_hACE2_ + G_spikeRBD_)

where G_hACE2-spikeRBD_ is the free energy of the hACE2-spikeRBD complex, G_hACE2_ is the free energy of the hACE2 and G_spikeRBD_ is the free energy of the spike RBD protein. ∆G_bind_ is calculated for each mutated spike RBD in this way.

### 4.4. MD-Based Alchemical Free Energy Calculations

**Figure 8:**
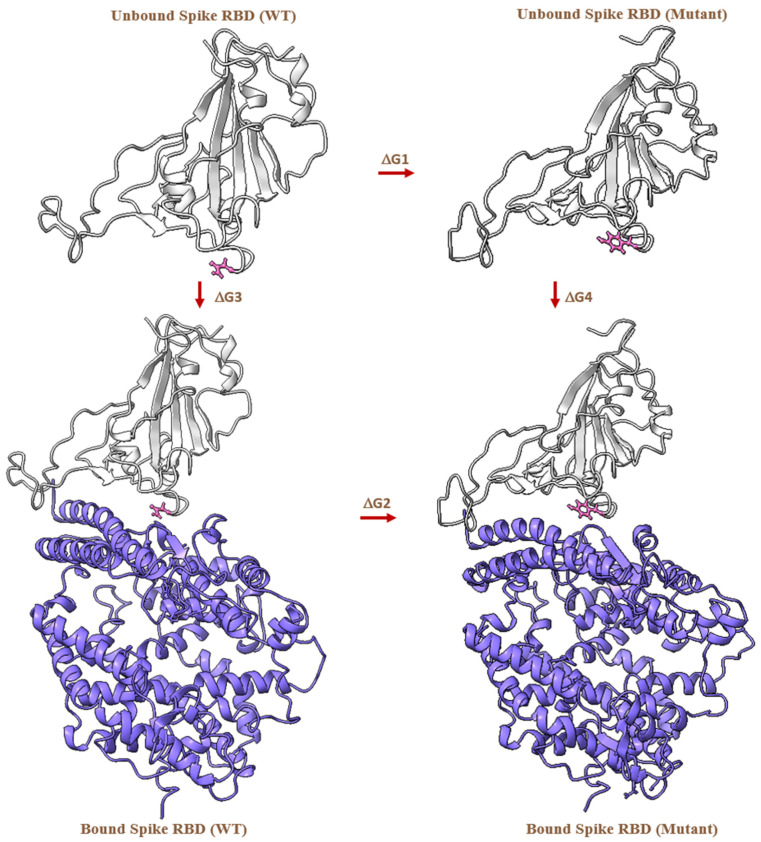
Alchemical free energy cycle represented for the SARS-CoV-2 spike RBD (WT and mutant) in an unbound and bound state with hACE2. The arrows indicate the free energy cycle. Asn-501 in WT and Tyr-501 in the mutant structure are shown in pink ball and stick representation, spike RBD in gray and hACE2 in purple cartoon representation. (The free energy cycle is repeated in same way for all the other mutations).

Alchemical relative free energy calculations exploit and avoid the need to simulate binding and unbinding events by making use of the fact that the free energy is a state function, and can be explained as in Equation (1) [106,107].

(1) $$\begin{array}{l} \Delta \Delta G_{bind,AB}\approx -k_{B}T\left(In\frac{Z\left(RB\right)}{Z\left(RA\right)}-In\frac{Z\left(R+B\right)}{Z\left(R+A\right)}\right)\\ \;=-k_{B}T\left(In\frac{Z\left(RB\right)}{Z\left(RA\right)}-In\frac{Z\left(B\right)}{Z\left(A\right)}\right)\\ \;=\Delta G_{bound}-\Delta G_{unbound} \end{array}$$

where $\Delta G_{bound}$ and $\Delta G_{unbound}$ represent the free energy of bound and unbound state, respectively. The unbound state is independent of the presence of the receptor in the simulations. The thermodynamic cycle [88] is illustrated in Figure 8.

According to Equation (1) and Figure 6, the difference in the free energy, for example, for the N501Y mutation, can be calculated by performing two independent MD simulations and estimating the free energy change from Asn-501 to Tyr-501 in the presence or absence of hACE2, i.e., ($\Delta G_{bound}$) and ($\Delta G_{unbound})$, respectively. In an alchemical simulation, this change from state A to state B is performed by introducing an alchemical progress parameter, lambda ($\vec{l}$), which controls the potential energy function $U(\vec{q};\vec{l}$) (*q* is the x,y,z coordinates of the simulation system). This is achieved by generating and simulating a hybrid topology of amino acids composed of the atoms that represent both A and B states. A subset of the energetic terms in $U(\vec{q};\vec{l}$) are modulated such that at ${\vec{l}}_{A},$ the atoms representing state A are activated and those representing state B are non-interacting “dummy atoms,” and vice versa at ${\vec{l}}_{B}$. In this way, the alchemical transformation from state A to B and vice versa is performed, and the free energy of mutating A to B in any environment (e.g., bound or unbound state) is computed as per the following Equation (2) [61,88].

(2) $$\Delta G_{env}=-k_{B}T\;In\frac{Z{\vec{(l}}_{B})}{Z{\vec{(l}}_{A})}=-k_{B}T\;In\frac{\int G_{env}\left(U\left(\vec{q;}{\vec{l}}_{B}\right)\right)\;d\vec{q}}{\int G_{env}\left(U\left(\vec{q;}{\vec{l}}_{A}\right)\right)\;d\vec{q}}$$

The derivatives of the Hamiltonian with respect to λ were recorded at every step, and free energies were calculated from the work (*W*) distributions obtained from integration, as per the Equation (3) [107].

(3) $$W=\int_{l=0}^{l=1}\frac{dH}{dl}dl$$

Soft-core potentials were used for both electrostatics and Lennard-Jones interactions as implemented in GROMACS. Finally, ΔΔG was estimated for each mutation by calculating the intersection of the forward and backward work distributions according to the Bennet’s Acceptance Ratio (BAR) method [108] (see Supporting Information, Figures S8–S13).

### 4.5. Preparation of Hybrid Topology

The PMX webserver [109,110] was used to generate the hybrid topology files for the selected amino acids. The pdb2gmx command was executed to reassign correct hydrogen atoms in the input coordinate files. The Amber99SB force field was used [64,111], and the output files containing the topology information were generated in a Gromacs readable form.

### 4.6. Equilibrium and Non-Equilibrium MD Simulations

All MD simulations (equilibrium and non-equilibrium) were carried out on the Puhti HPC cluster [112] with GROMACS-2020.5 [113,114,115,116,117,118]. The hybrid topologies generated by the PMX webserver were used as an input. For each mutation, we prepared an individual simulation box and performed forward and backward transition with two simulation systems representing the protein in state A and state B (λ= 0 and λ = 1) (see the input scripts in Supporting Information, pp. S12–S15). The Amber99SB force field and the Joung and Cheatham ion parameters were used [119]. Each state was solvated using a dodecahedral water box with a simple point charge 216 (SPC216)102 3-point water model, and neutralized with Na^+^ and Cl^−^ ions at a 0.15 M concentration. Each simulation system was then energy-minimized for 100,000 steps using the steepest descent algorithm [120]. This was followed by 1-ns NPT ensemble simulation with positional restraints, using a stochastic leap-frog integrator and isotropic Berendsen pressure coupling [121]. The final production MD simulations were performed using the stochastic leap-frog integrator for 50 ns [122]. All bond lengths were constrained using the LINCS algorithm [123]. The constant pressure and temperature condition was maintained using the Parrinello−Rahman pressure coupling [124] at 1 atm and temperature coupling at 298.15 K. A 2-fs time step was used, and the trajectory was recorded at every 10 ps. In order to treat the long-range electrostatics, the Particle Mesh Ewald (PME) method with a direct space cut-off of 1.1 nm and Fourier grid spacing of 0.12 nm were used [125,126]. The relative strength of the Ewald-shifted direct potential was set to 10^−5^. Van der Waals interactions were smoothly switched off between 1.0 and 1.1 nm.

In order to perform the alchemical transformations after the equilibrium MD simulations, fast-growth non-equilibrium simulations were performed in order to estimate the binding free energy (ΔΔG) value [61,64,107]. For this, each equilibrium simulation was used as input for a corresponding non-equilibrium run; the first 25 ns of the trajectory were omitted, and the last 25 ns of the simulation were used to generate 50 snapshots from every 500 ps. The non-equilibrium simulation was performed for a total of 5 ns, with the lambda value changing continuously in forward and reverse direction from state 0 to 1, and vice versa, at each time step with a frequency of transition of 2 × 10^−7^. The standard errors of the ΔG estimates were obtained by bootstrapping and reflect the variability in the datasets (trajectories) analyzed.

## Figures and Tables

**Table 1:** Selected mutations for the study.

Mutation	Identified in Variants	Increases hACE2 Binding	Evading Antibodies	Experimental Binding Affinity of Spike Glycoprotein to hACE2 (K_D_ in nM)
G446S	O (BA.1)	No [66,67]	Yes [68]	46.9 [66]
F486V	O (BA.4, BA.5)	No [69,70,71]	Yes [72]	-
G496S	O (BA.1)	No [69]	Yes [68]	-
N501Y	β, α, γ, µ, θ, O	Yes [73,74]	Yes (slightly) [75]	2.4 ± 0.4 [74] 3.0 ± 2.1 [76] 5.5 ± 2.4 [40] 10.7 [77] 0.4 [78]
Y505H	O	No [69,70,71]	Yes [68]	-

N.B. Experimental binding affinity (Kd in nM) of WT spike glycoprotein to hACE2: 29.4 ± 2.62 [66], 17 ± 0.6 [74], 10 ± 3.1 [76], 62.6 ± 7.7 [40], 75.1 [77], 16 [78]. nM.—data not available.

**Table 2:** Binding free energies towards hACE2 for the studied SARS-CoV-2 spike RBD mutants obtained from alchemical free energy calculations.

Spike RBD Mutant	Bound ΔG (kJ/mol)	Unbound ΔG (kJ/mol)	ΔΔG (kJ/mol)	ΔΔG (kcal/mol)
G446S	1.80± 0.28	2.97 ± 0.09	−1.17 ± 0.29	−0.28 ± 0.07
F486V	−95.80 ± 0.27	−99.99 ± 0.18	4.19 ± 0.32	1.00 ± 0.08
G496S	24.99 ± 1.65	12.57 ± 0.70	12.42 ± 1.79	2.97 ± 0.43
N501Y	290.22 ± 0.84	304.24 ± 0.46	−14.02 ± 0.96	−3.35 ± 0.23
Y505Hɛ	−2.57 ± 0.44	−6.05 ± 0.20	3.48 ± 0.48	0.83 ± 0.12
Y505Hδ	2.87 ± 0.41	0.62 ± 0.14	2.25 ± 0.43	0.54 ± 0.10

## Data Availability

The data generated during the current study are available in the Zenodo repository with the DOI identifier 10.5281/zenodo.7263848.

## Supplementary Materials

The following supporting information can be downloaded at: https://www.mdpi.com/article/10.3390/biomedicines10112779/s1, Figure S1: The averaged RMSD of the WT and mutated protein structures of the spike RBD in complex with hACE2 during three replicates of 200-ns MD simulations; Figure S2: The RMSD of the Y505HIE mutant protein structure of the spike RBD in complex with hACE2 during three replicates of 300-ns MD simulations; Figure S3: The averaged RMSF of the WT and mutated protein structures of spike RBD in complex with hACE2 during three replicates of 200-ns MD simulations; Figure S4: Averaged RMSF of the residues surrounding the individual mutations of spike RBD in complex with hACE2 during three replicates of 200-ns MD simulations; Figure S5: Number of pi-pi stacking interactions between Phe486 of the wildtype spike RBD and Tyr83 of hACE2 during a 200-ns MD simulation; Figure S6: Number of pi-pi stacking interactions between Tyr501 of the mutant spike RBD and Tyr41 of hACE2 during a 200-ns MD simulation; Figure S7: The aromatic ring distance between Tyr501 of the mutant spike RBD and Tyr41 of hACE2 during three independent 200-ns MD simulations; Figure S8: Free energy plots for unbound and bound G446S; Figure S9: Free energy plots for unbound and bound F486V; Figure S10: Free energy plots for unbound and bound G496S; Figure S11: Free energy plots for unbound (top) and bound (bottom) N501Y; Figure S12: Free energy plots for unbound and bound Y505HID; Table S1: Experimental binding affinities of SARS-CoV-2 spike RDB mutants converted to change in binding free energies; Page S19: Input scripts for alchemical free energy calculations.
