# Supplementary material for: High-Throughput Molecular Dynamics-Based Alchemical Free Energy Calculations for Predicting the Binding Free Energy Change Associated with the Selected Omicron Mutations in the Spike Receptor-Binding Domain of SARS-CoV-2
Source: Biomedicines. 2022 Nov 1;10(11):2779. doi: 10.3390/biomedicines10112779 (PMC9687918; doi:10.3390/biomedicines10112779)
Supplement: Supplementary file 1 [file biomedicines-10-02779-s001.zip › biomedicines-1908766-supplementary.pdf]

**Supporting Information**

# High-throughput molecular dynamics-based alchemical free energy calculations for predicting the binding free energy change associated with the selected omicron mutations in the spike receptor-binding domain of SARS-CoV-2

**Rajendra Bhadane[1,2], Outi M. H. Salo-Ahen[1,2]**

[1]*Structural Bioinformatics Laboratory, Faculty of Science and Engineering, Biochemistry, Åbo Akademi University, FI-20520 Turku, Finland*

[2]*Pharmaceutical Sciences Laboratory, Faculty of Science and Engineering, Pharmacy, Åbo Akademi University, FI-20520 Turku, Finland*

**Correspondence:*

*Rajendra Bhadane Tel + 358-2-2154006; Email: rajendra.bhadane@abo.fi*

*Outi M. H. Salo-Ahen Tel +358-2-215 4009; Email: outi.salo-ahen@abo.fi*

**Results of conventional MD simulations:**

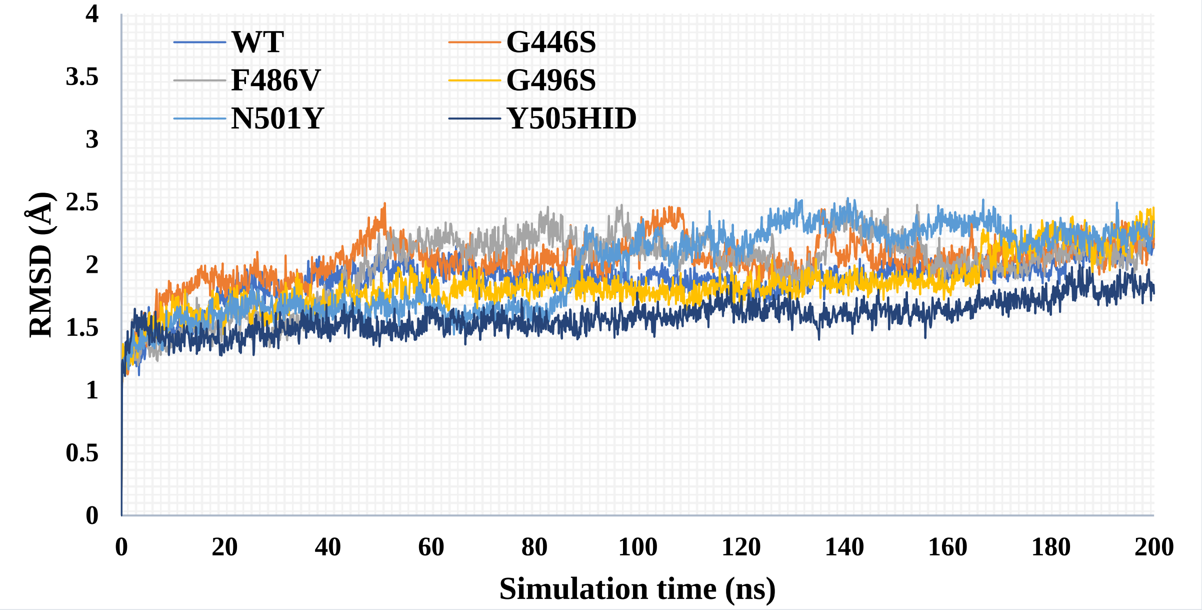


***Figure S1.*** *The* ***averaged*** *root-mean-square deviation (RMSD) of the wildtype (WT) and mutated protein structures of the spike RBD in complex with hACE2 during* ***three replicates*** *of* ***200-ns*** *MD simulations. HID – histidine protonated at the delta nitrogen.*

**The RMSD plots for each individual MD run in Figure S1 are shown here below for WT and on pages S3-S5 for the mutants:**

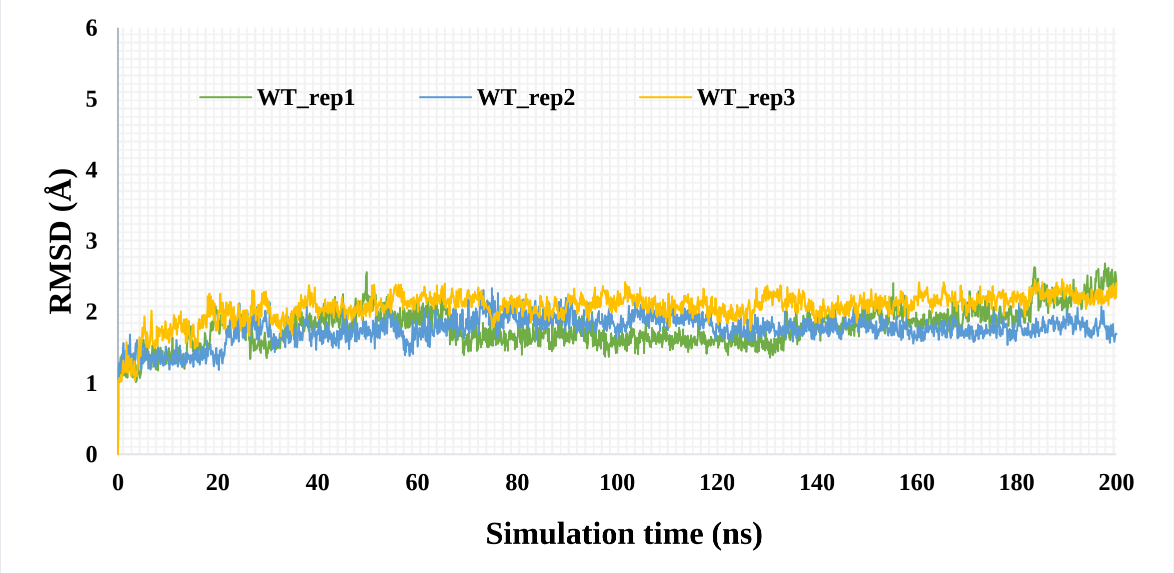

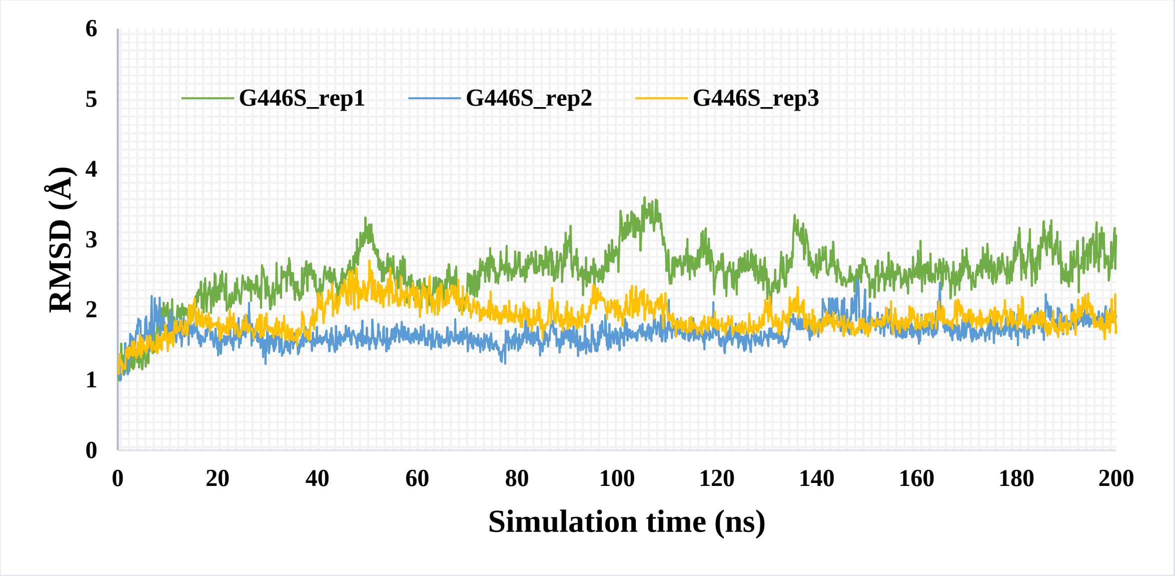
G446S_rep1
G446S_rep2
G446S_rep3
RMSD (Å)
Simulation time (ns)

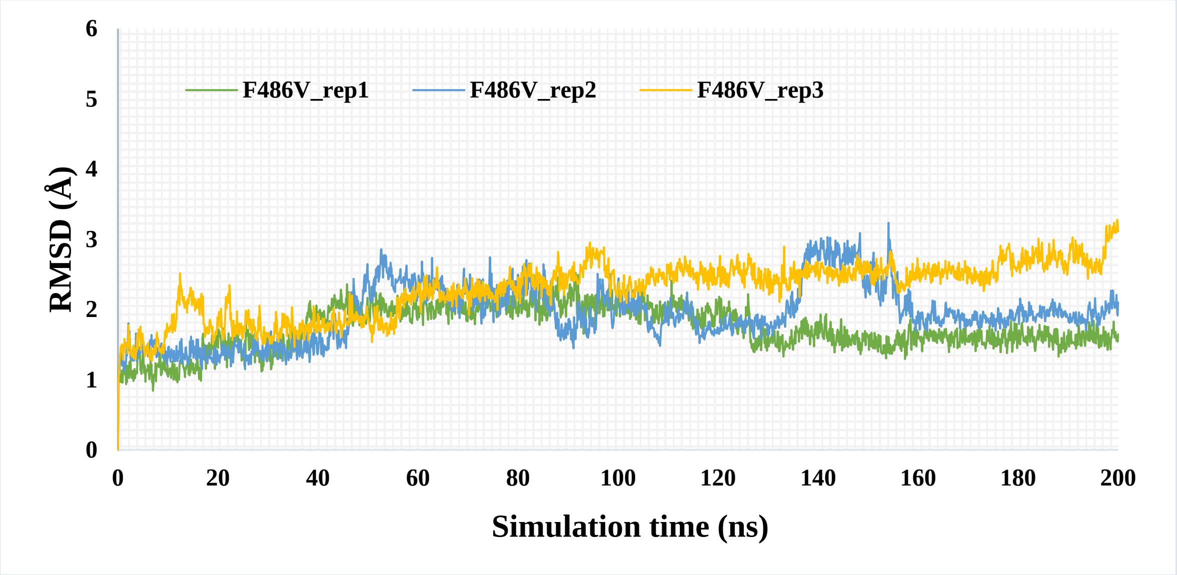
F486V_rep1
F486V_rep2
F486V_rep3
RMSD (Å)
Simulation time (ns)

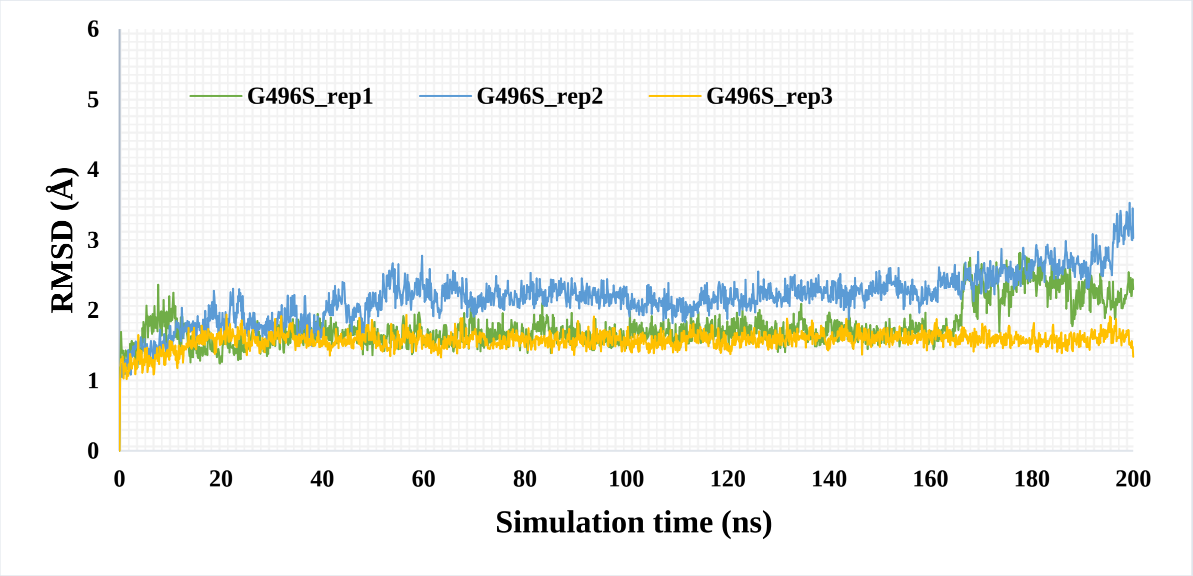
G496S_rep1
G496S_rep2
G496S_rep3
RMSD (Å)
Simulation time (ns)

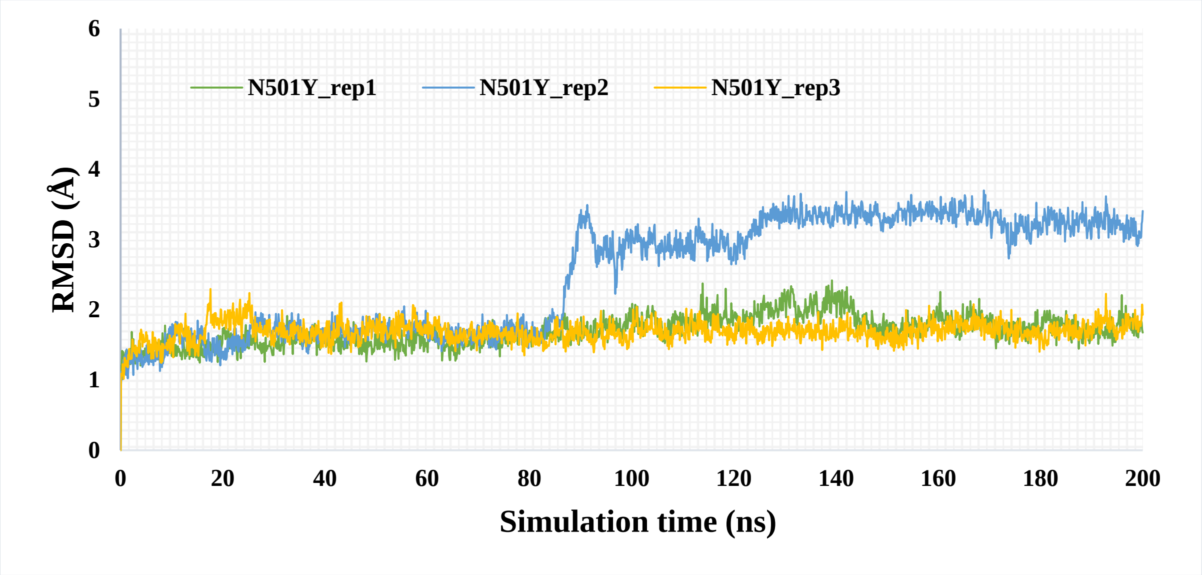

N501Y_rep1
N501Y_rep2
N501Y_rep3
RMSD (Å)
Simulation time (ns)


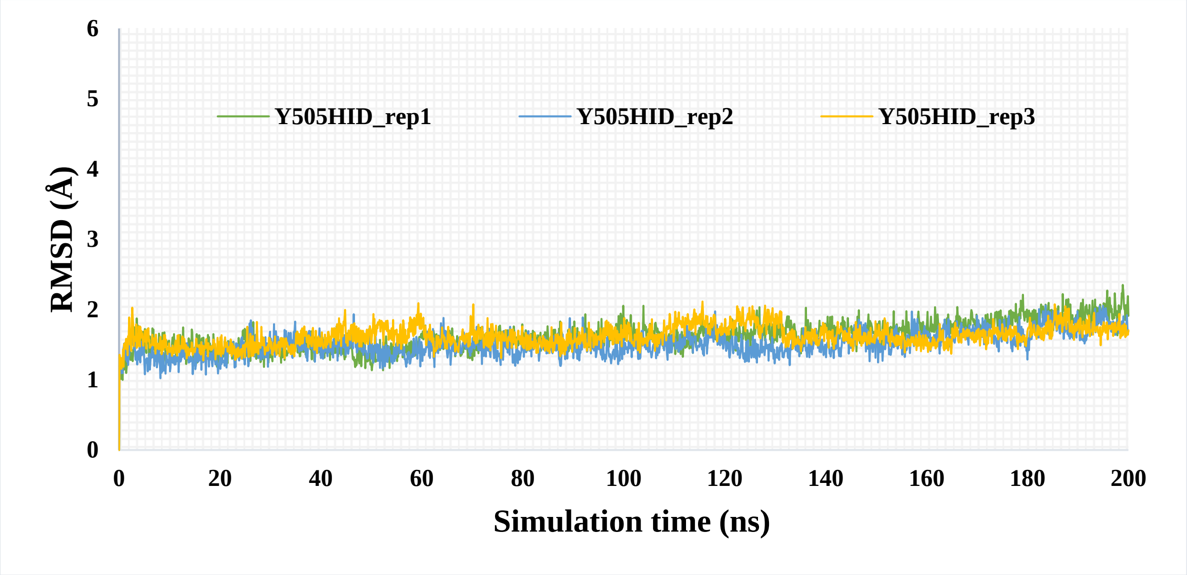

Y505HID_rep1
Y505HID_rep2
Y505HID_rep3
RMSD (Å)
Simulation time (ns)

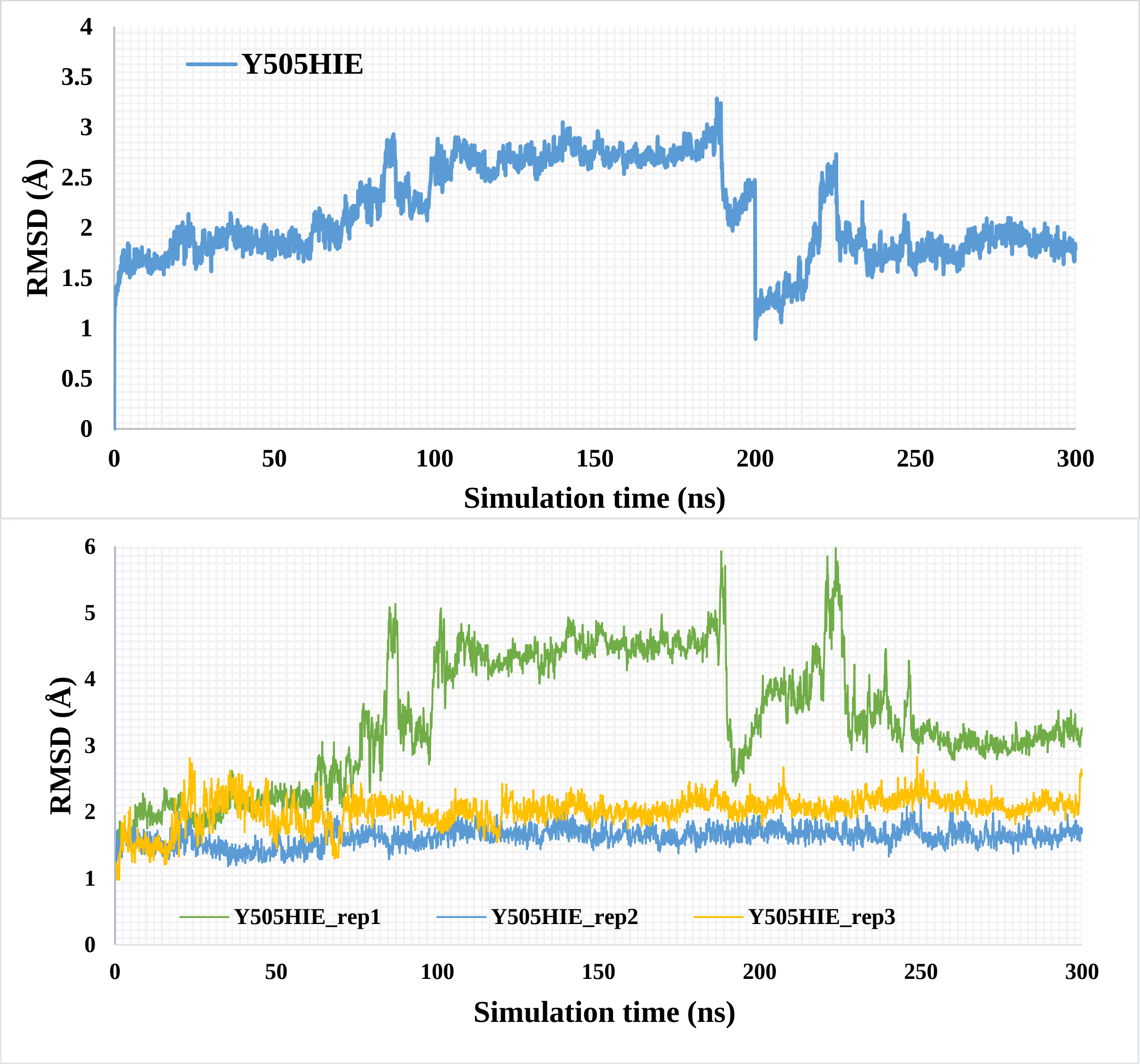


***Figure S2.*** *The root-mean-square deviation (RMSD) of the* ***Y505HIE*** *mutant protein structure of the spike RBD in complex with hACE2 during three replicates of* ***300-ns*** *MD simulations. Top: average of three simulations. Bottom: RMSD for the individual simulations. HIE – histidine protonated at the epsilon nitrogen.*

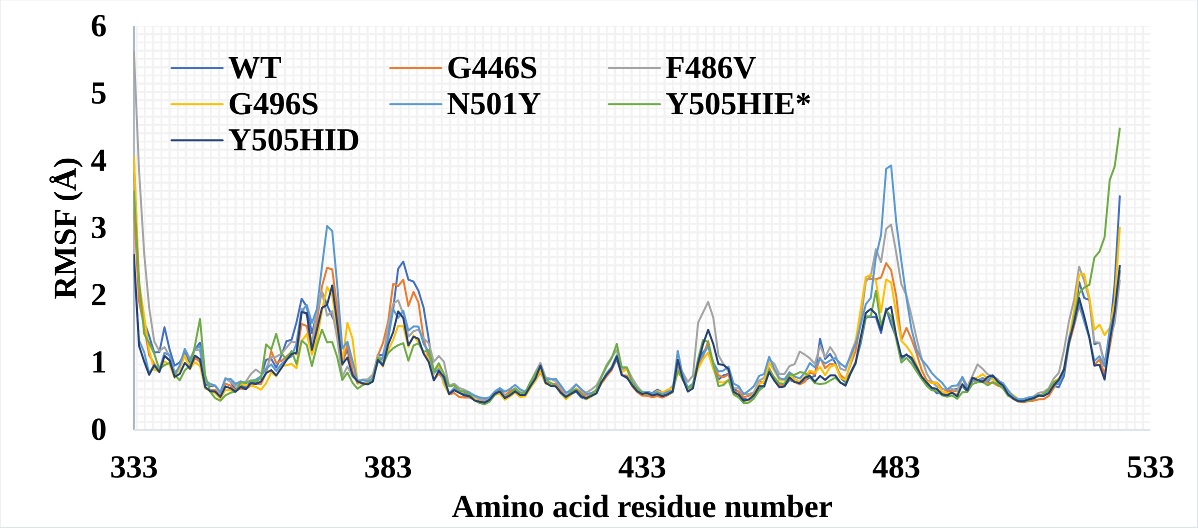


***Figure S3.*** *The averaged root-mean-square fluctuation (RMSF) of the WT and mutated protein structures of spike RBD in complex with hACE2 during three replicates of 200-ns MD simulations (*Y505HIE mutant was simulated for 300 ns). HID – histidine protonated at the delta nitrogen; HIE – histidine protonated at the epsilon nitrogen.*

**The RMSF plots for each individual MD run are shown here below for WT and on pages S7 and S8 for the mutants:**

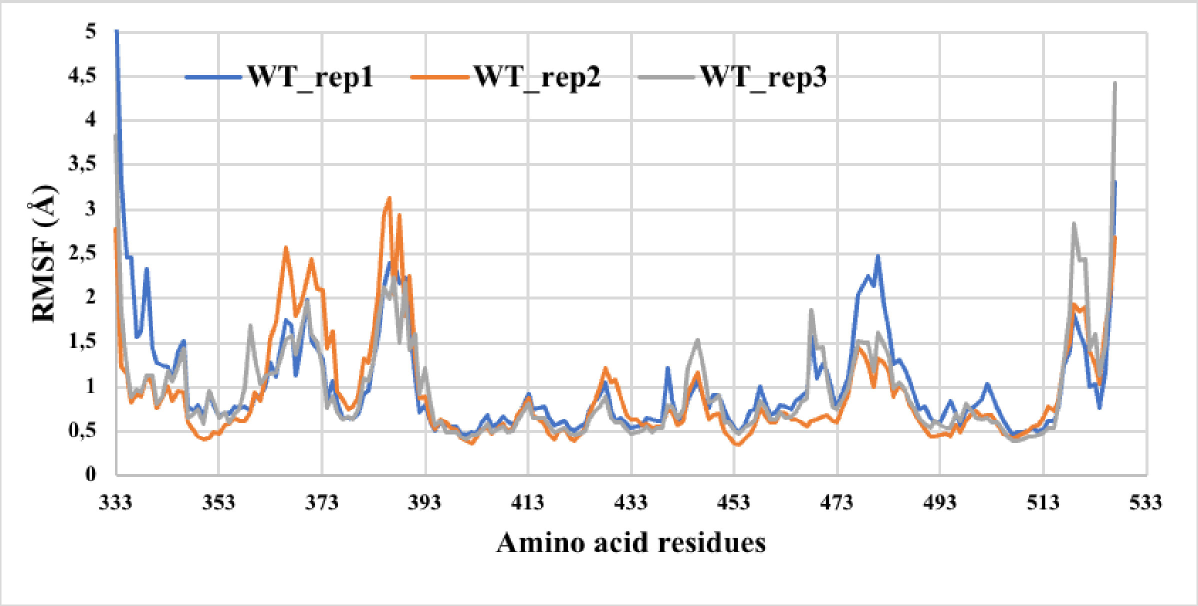

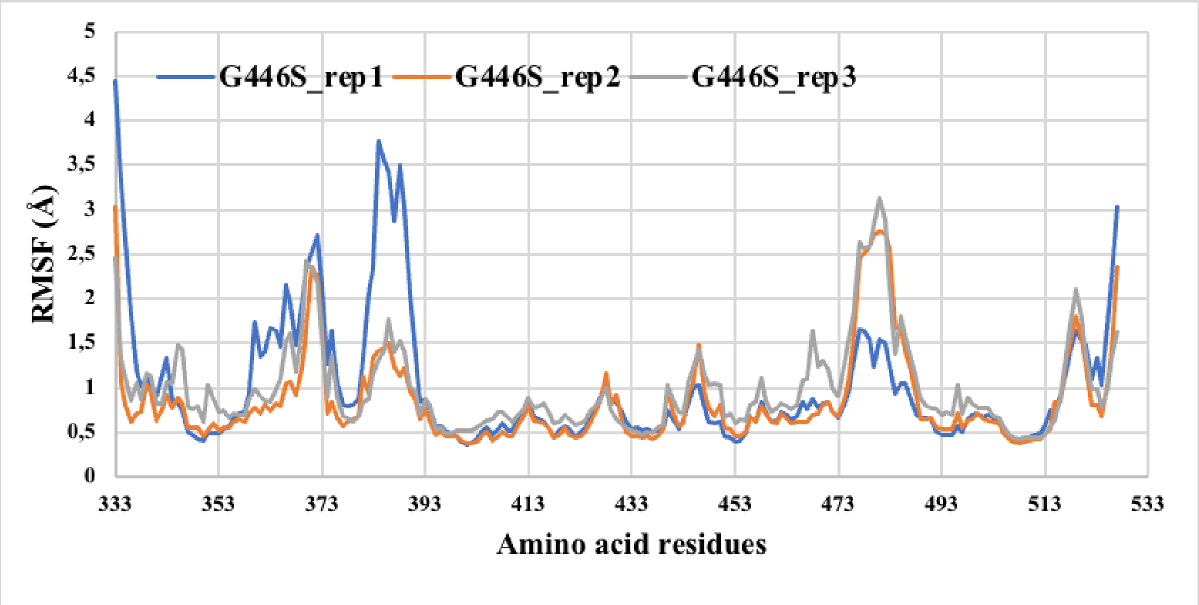

G446S_rep1
G446S_rep2
G446S_rep3
RMSF (Å)
Amino acid residues


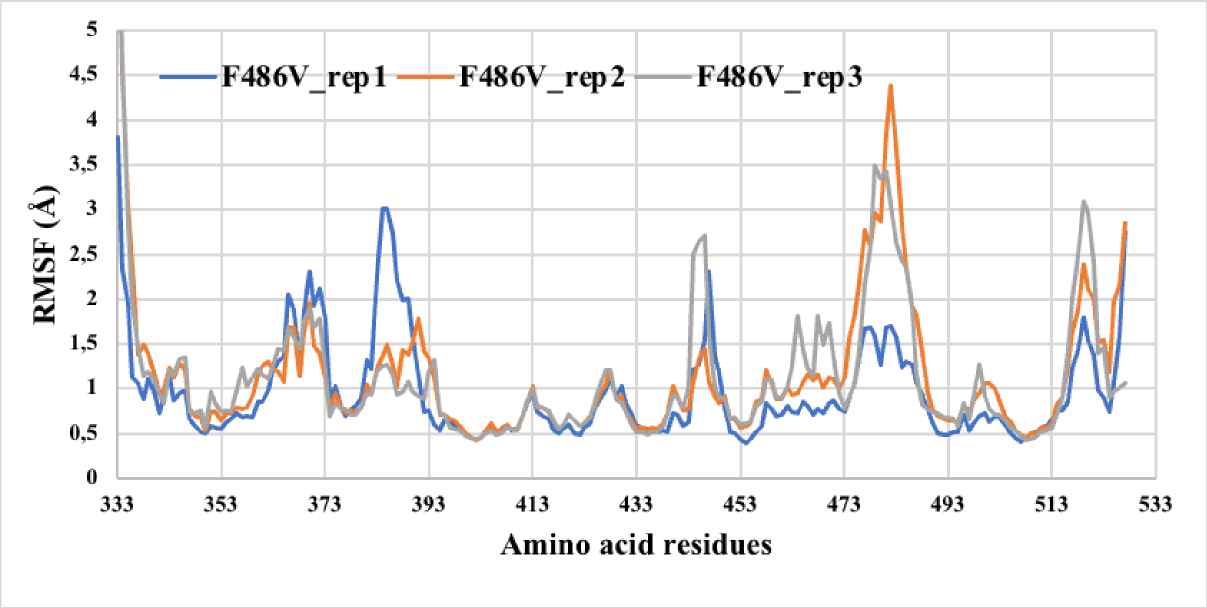

F486V_rep1
F486V_rep2
F486V_rep3
RMSF (Å)
Amino acid residues


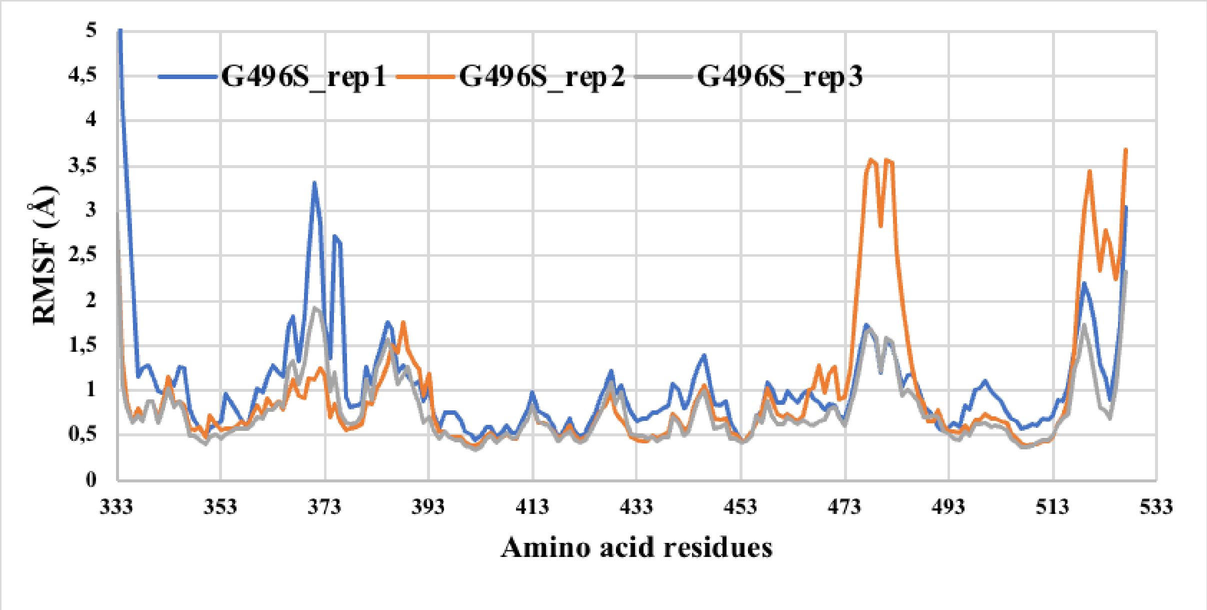

G496S_rep1
G496S_rep2
G496S_rep3
RMSF (Å)
Amino acid residues

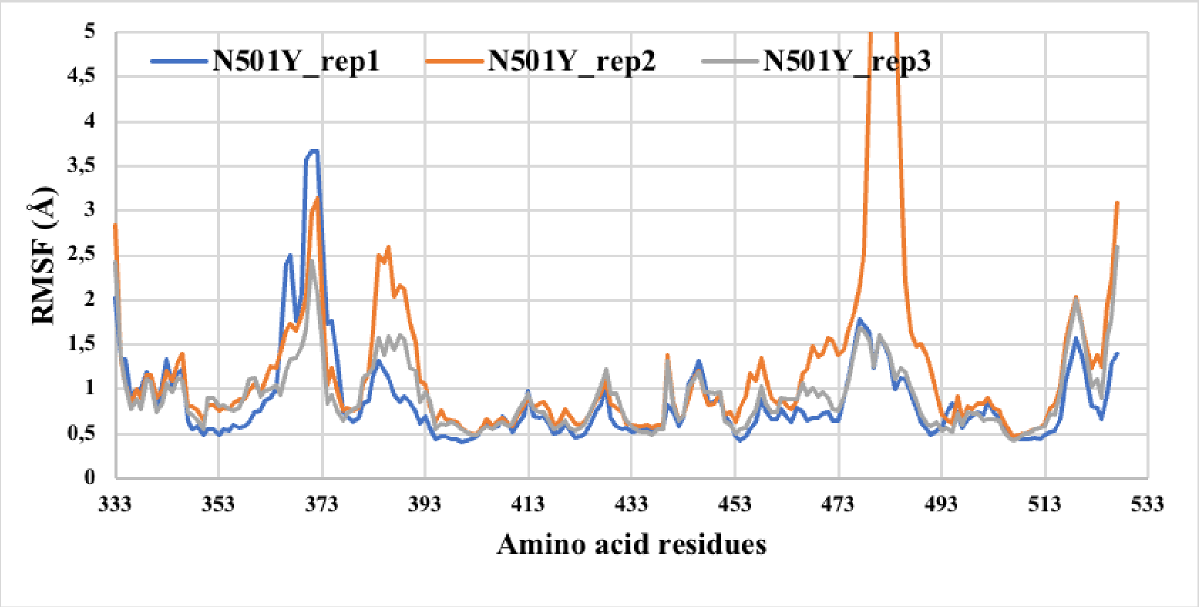

N501Y_rep1
N501Y_rep2
N501Y_rep3
RMSF (Å)
Amino acid residues


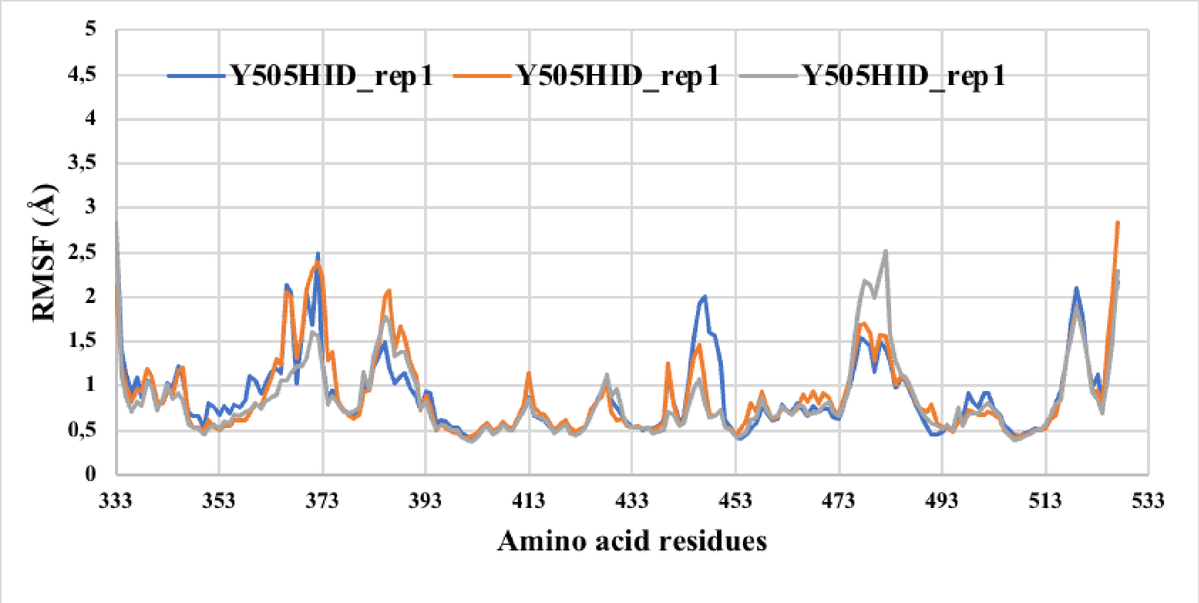

Y505HID_rep1
Y505HID_rep1
Y505HID_rep1
RMSF (Å)
Amino acid residues


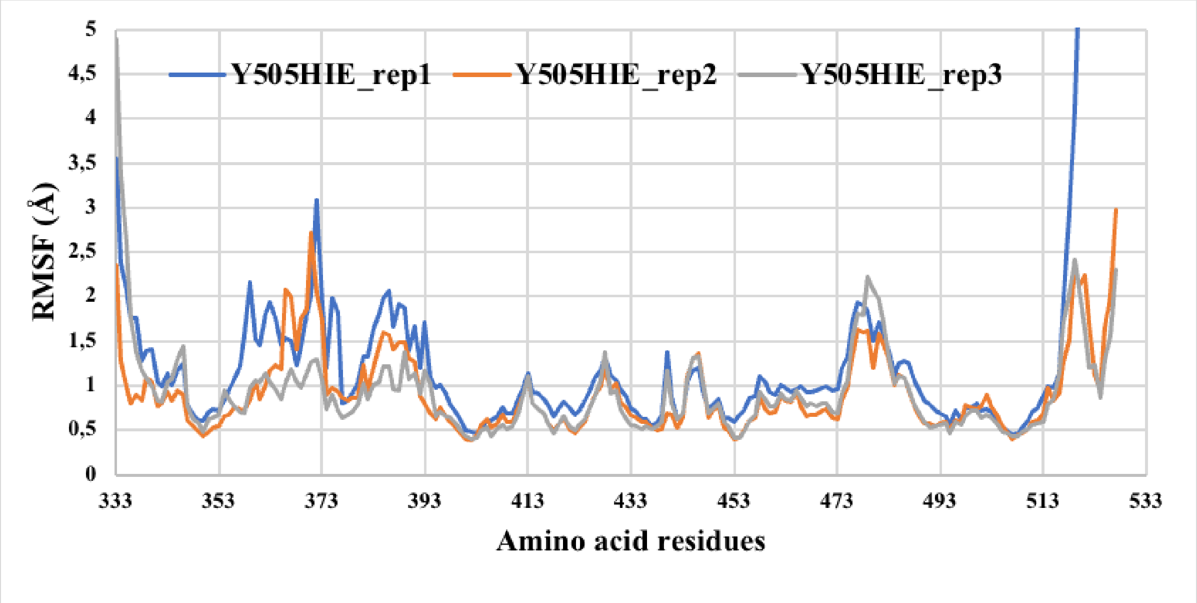

Y505HIE_rep1
Y505HIE_rep2
Y505HIE_rep3
RMSF (Å)
Amino acid residues

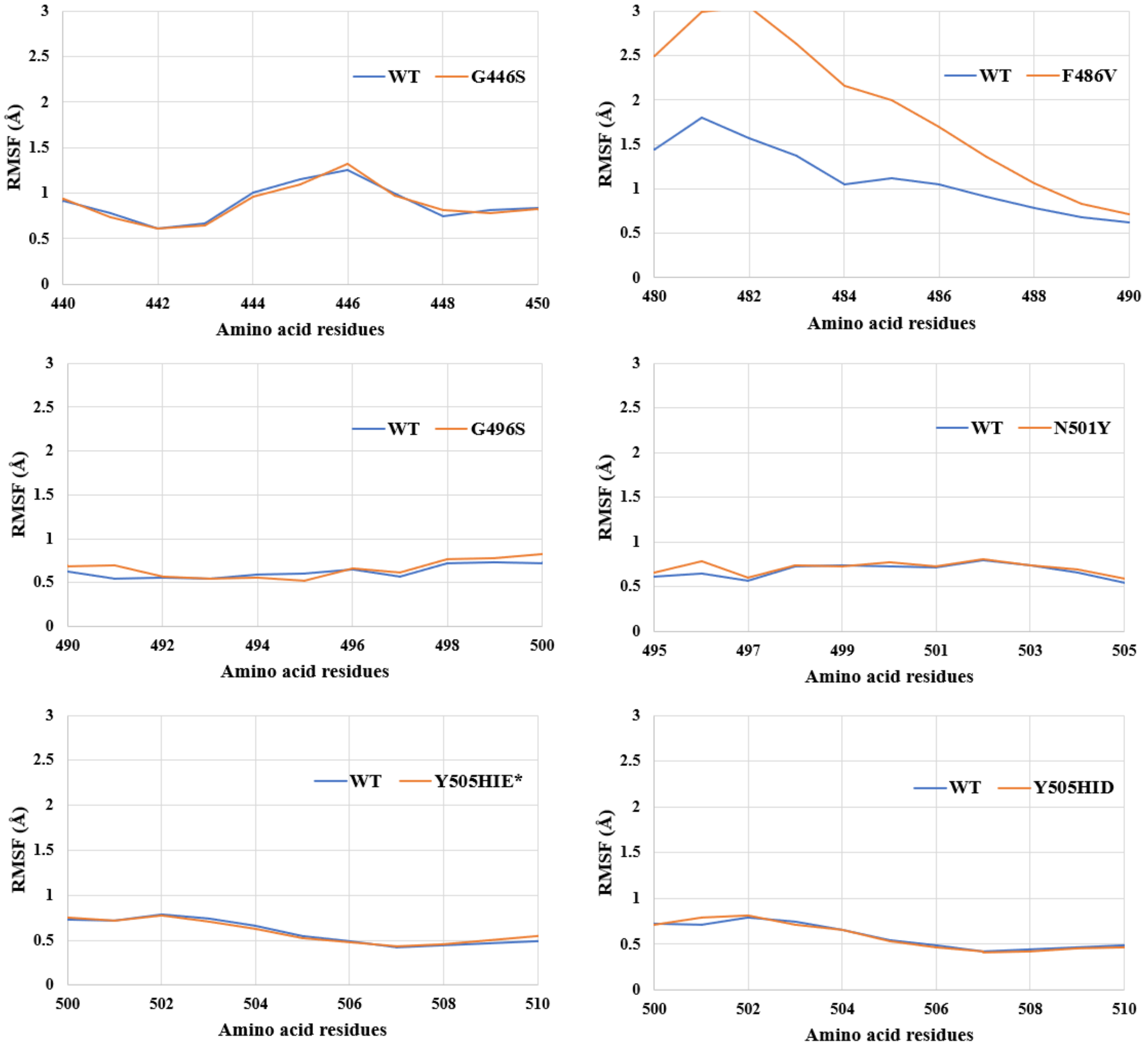


***Figure S4.*** *Averaged root-mean-square fluctuation (RMSF) of the residues surrounding the individual mutations of spike RBD in complex with hACE2 during three replicates of 200-ns MD simulations (*Y505HIE mutant was simulated for 300 ns) compared with the RMSF of the corresponding residues in the WT complex. HID – histidine protonated at the delta nitrogen; HIE – histidine protonated at the epsilon nitrogen.*

**Interaction analysis of spike RBD wildtype/mutant residues with hACE2 using conventional MD**

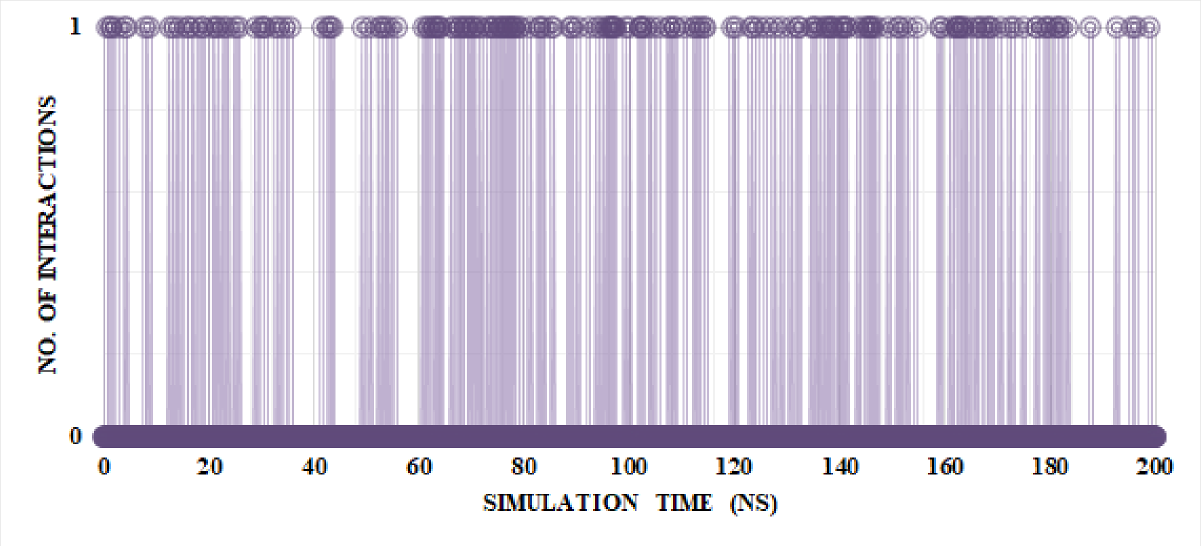


***Figure S5.*** *Number of pi-pi stacking interactions between Phe486 of the wildtype spike RBD and Tyr83 of hACE2 during a 200-ns MD simulation (replica 3).*

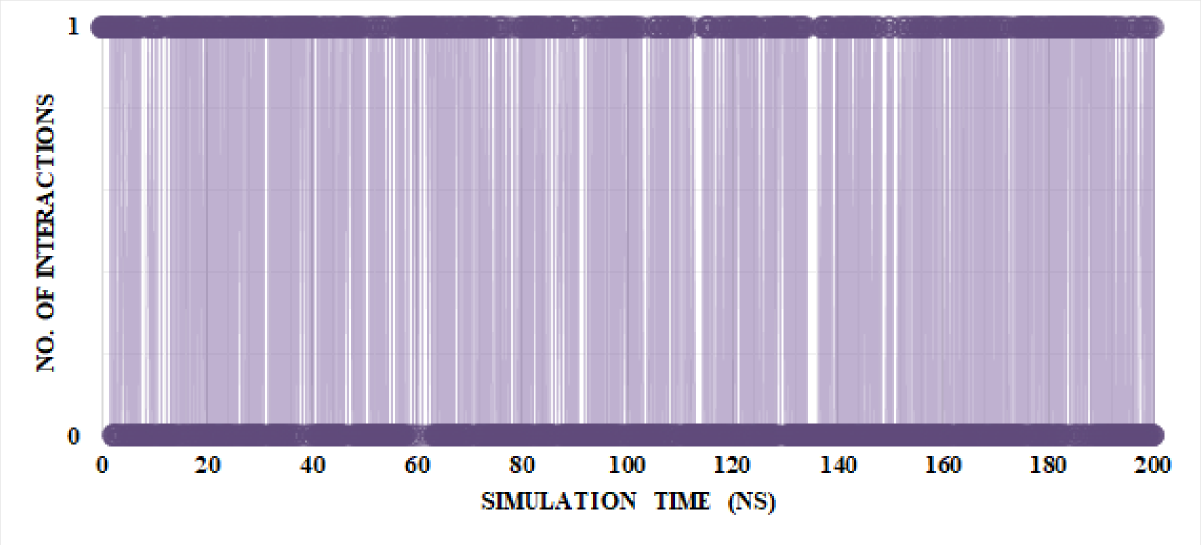


***Figure S6.*** *Number of pi-pi stacking interactions between Tyr501 of the mutant spike RBD and Tyr41 of hACE2 during a 200-ns MD simulation (replica 3).*

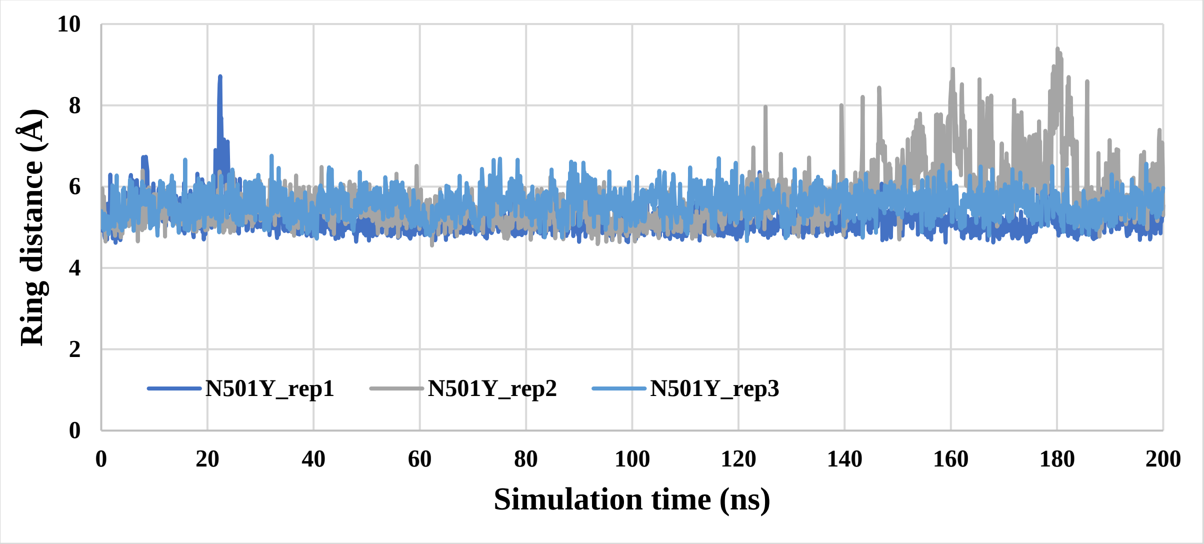


***Figure S7.*** *The aromatic ring distance between Tyr501 of the mutant spike RBD and Tyr41 of hACE2 during three independent 200-ns MD simulations.*

***Table S1.*** *Experimental binding affinities of SARS-CoV-2 spike RDB mutants converted to change in binding free energies (ΔΔG)*

| SARS-CoV-2 spike RBD mutation | ***$Kd_{MUT}$ (nM)*** | ***$Kd_{WT}$ (nM)*** | ***ΔΔG (kcal/mol)*** | ***Reference*** |
|---|---|---|---|---|
| **G446S** | *46.9±3.18* | *29.4±2.62* | *0.28±0.12* | *(66)* |
| **N501Y** | *2.4±0.4* | *17±0.6* | *-1.17±0.24* | *(74)* |
| | *3.0±2.1* | *10±3.1* | *-0.72±0.23* | *(76)* |
| | *5.5±2.4* | *62.6±7.7* | *-1.45±0.69* | *(40)* |
| | *10.7* | *75.1* | *-1.16* | *(77)* |
| | *0.40* | *16.0* | *-2.20* | *(78)* |

*Note: The experimental Kd values were taken from the studies collected in Table 1 and converted to ΔG (kcal/mol) using equation ΔG=RTlnKd, where R is gas constant (1.9872 mol/cal) and T is temperature in Kelvin (300 K). The final ΔΔG was calculated using the equation* $\Delta\Delta G=\Delta G_{MUT}-\Delta G_{WT}$.

**Results of the alchemical free energy calculations – PMX standard output: Final free energy plots of the mutation free energy pipeline**

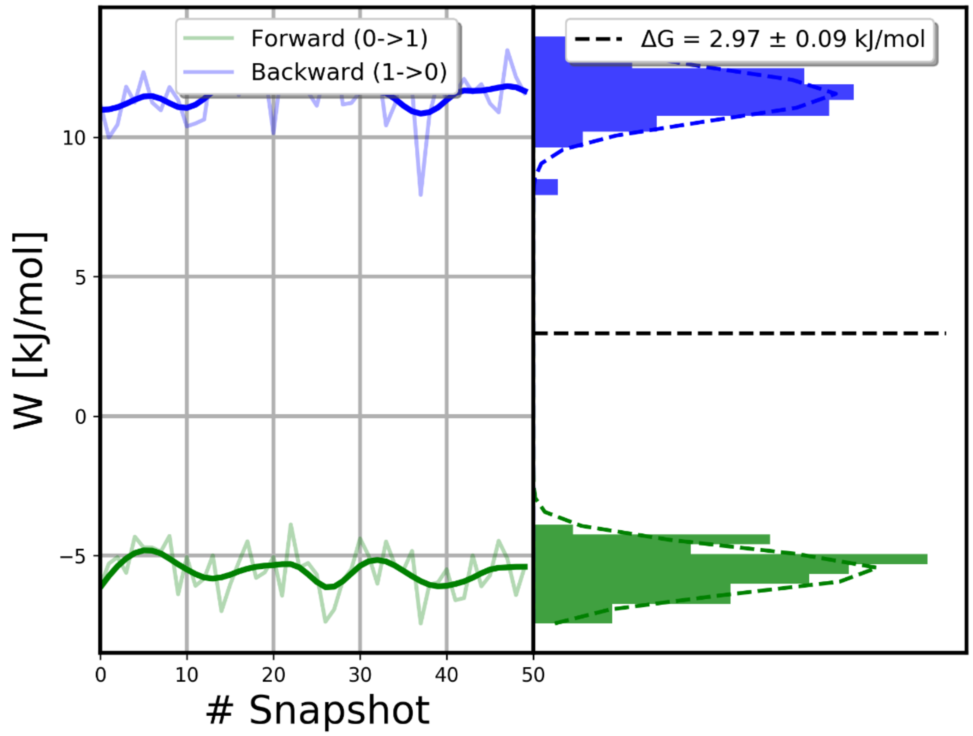


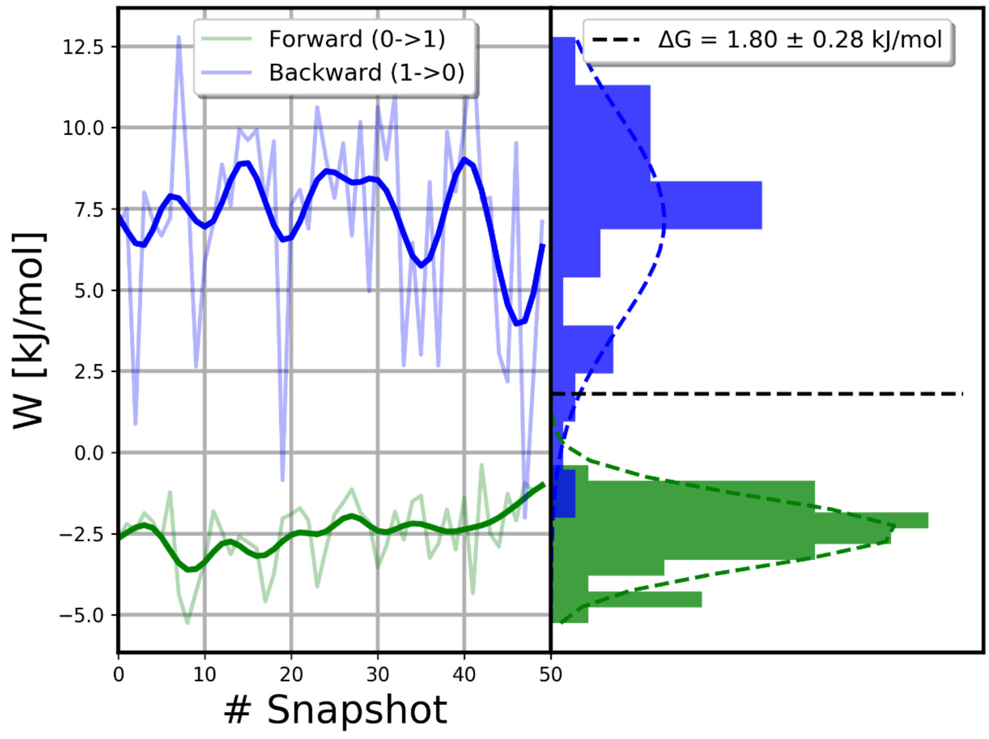


***Figure S8.*** *Free energy plots for unbound (top) and bound (bottom) G446S (each plot is the sum of 50 forward and 50 backward 5-ns non-equilibrium MD simulations).* ***Left:*** *Work values for the forward and backward transitions are plotted for every snapshot structure.* ***Right:*** *The work value distributions are depicted as histograms; their intersection gives the free energy difference.*

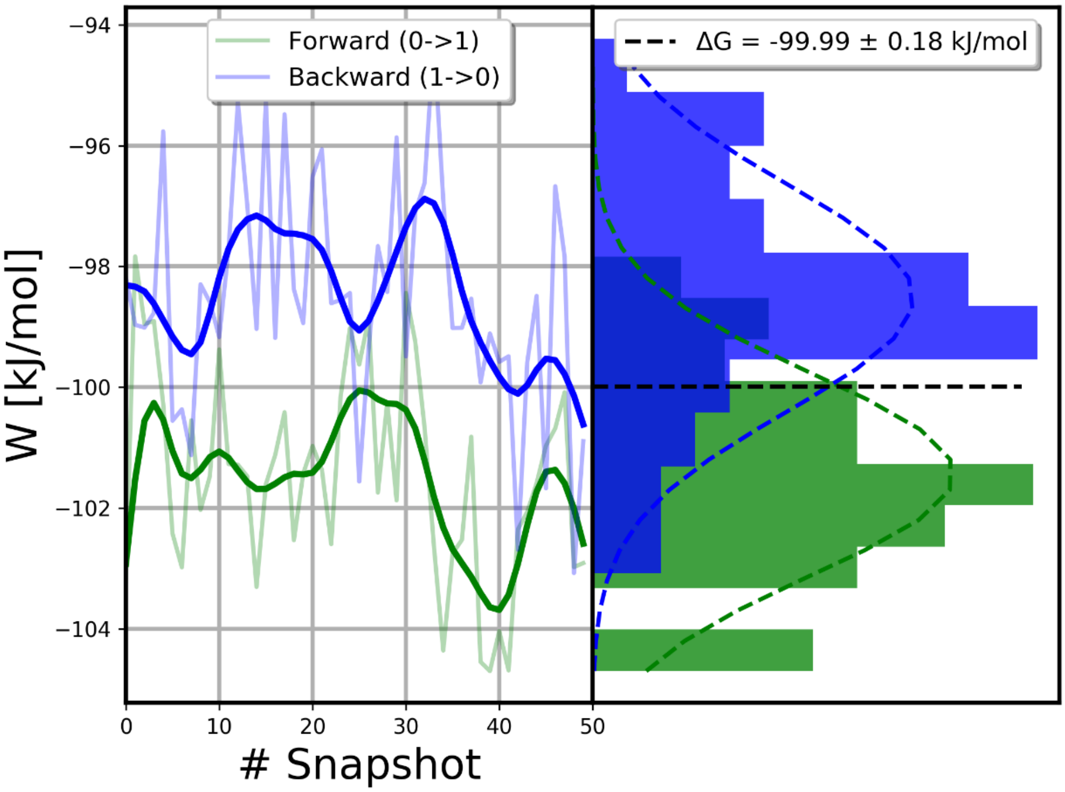


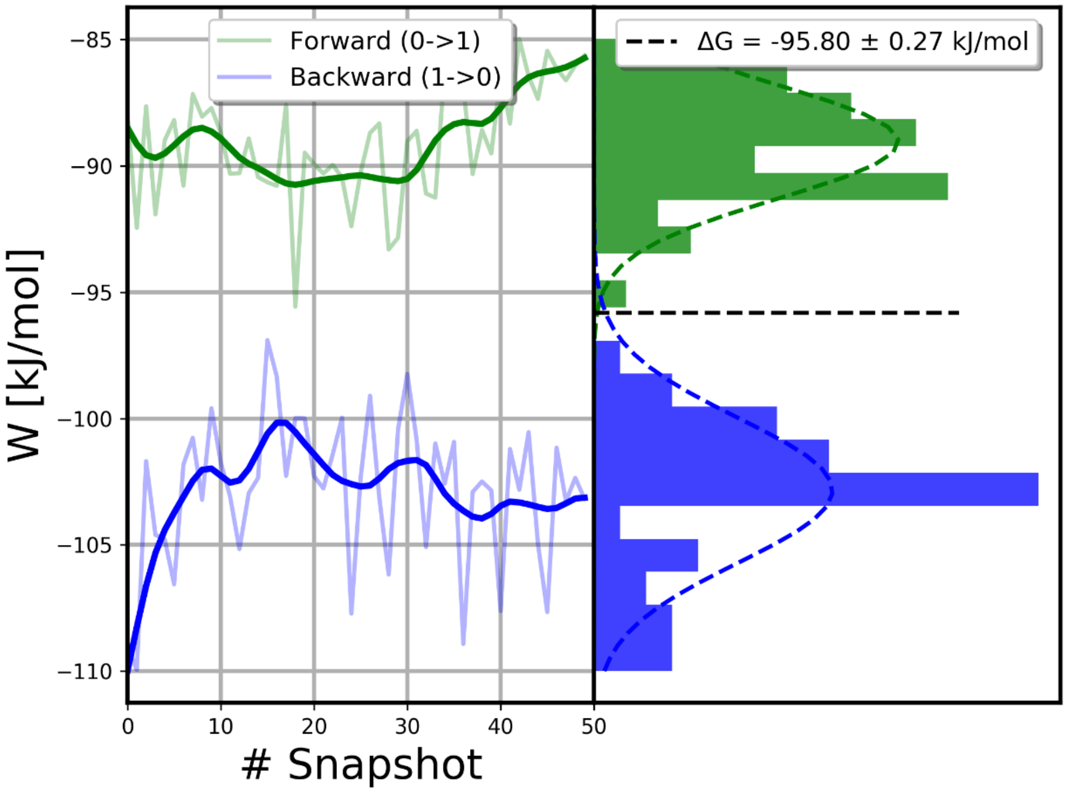


***Figure S9.*** *Free energy plots for unbound (top) and bound (bottom) F486V (each plot is the sum of 50 forward and 50 backward 5-ns non-equilibrium MD simulations).* ***Left:*** *Work values for the forward and backward transitions are plotted for every snapshot structure.* ***Right:*** *The work value distributions are depicted as histograms; their intersection gives the free energy difference.*

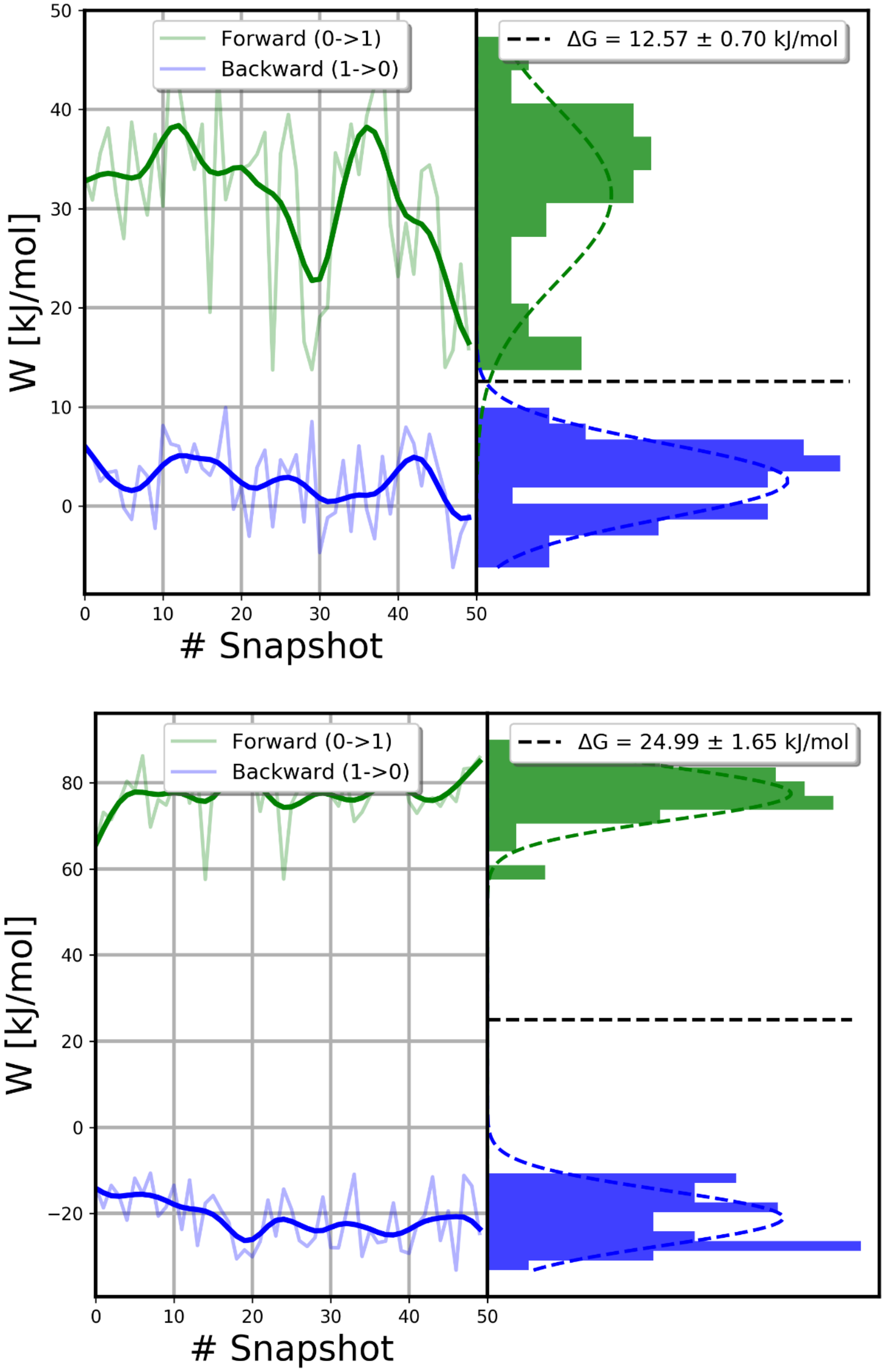


***Figure S10.*** *Free energy plots for unbound (top) and bound (bottom) G496S (each plot is the sum of 50 forward and 50 backward 5-ns non-equilibrium MD simulations).* ***Left:*** *Work values for the forward and backward transitions are plotted for every snapshot structure.* ***Right:*** *The work value distributions are depicted as histograms; their intersection gives the free energy difference.*

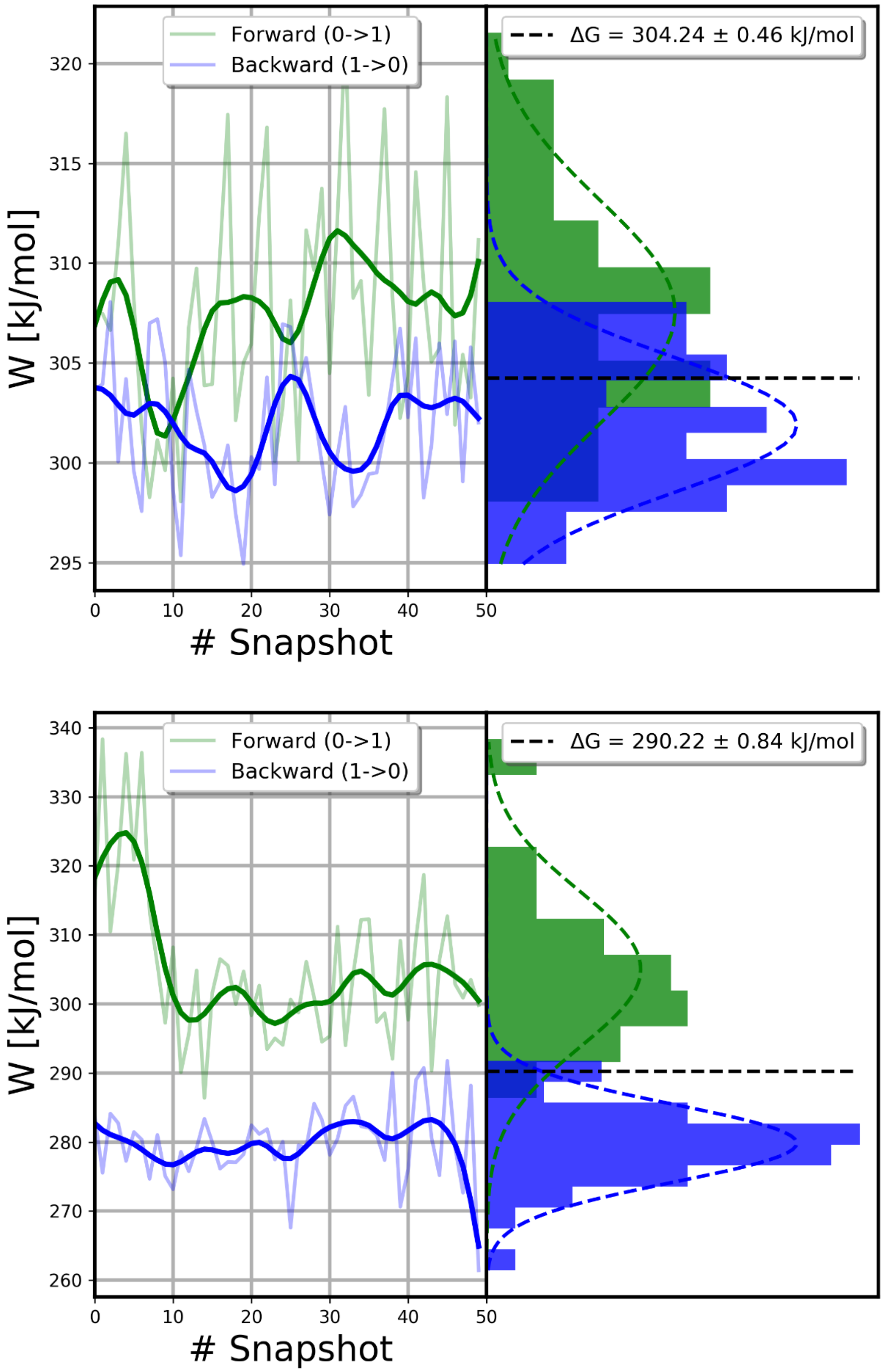


***Figure S11.*** *Free energy plots for unbound (top) and bound (bottom) N501Y (each plot is the sum of 50 forward and 50 backward 5-ns non-equilibrium MD simulations).* ***Left:*** *Work values for the forward and backward transitions are plotted for every snapshot structure.* ***Right:*** *The work value distributions are depicted as histograms; their intersection gives the free energy difference.*

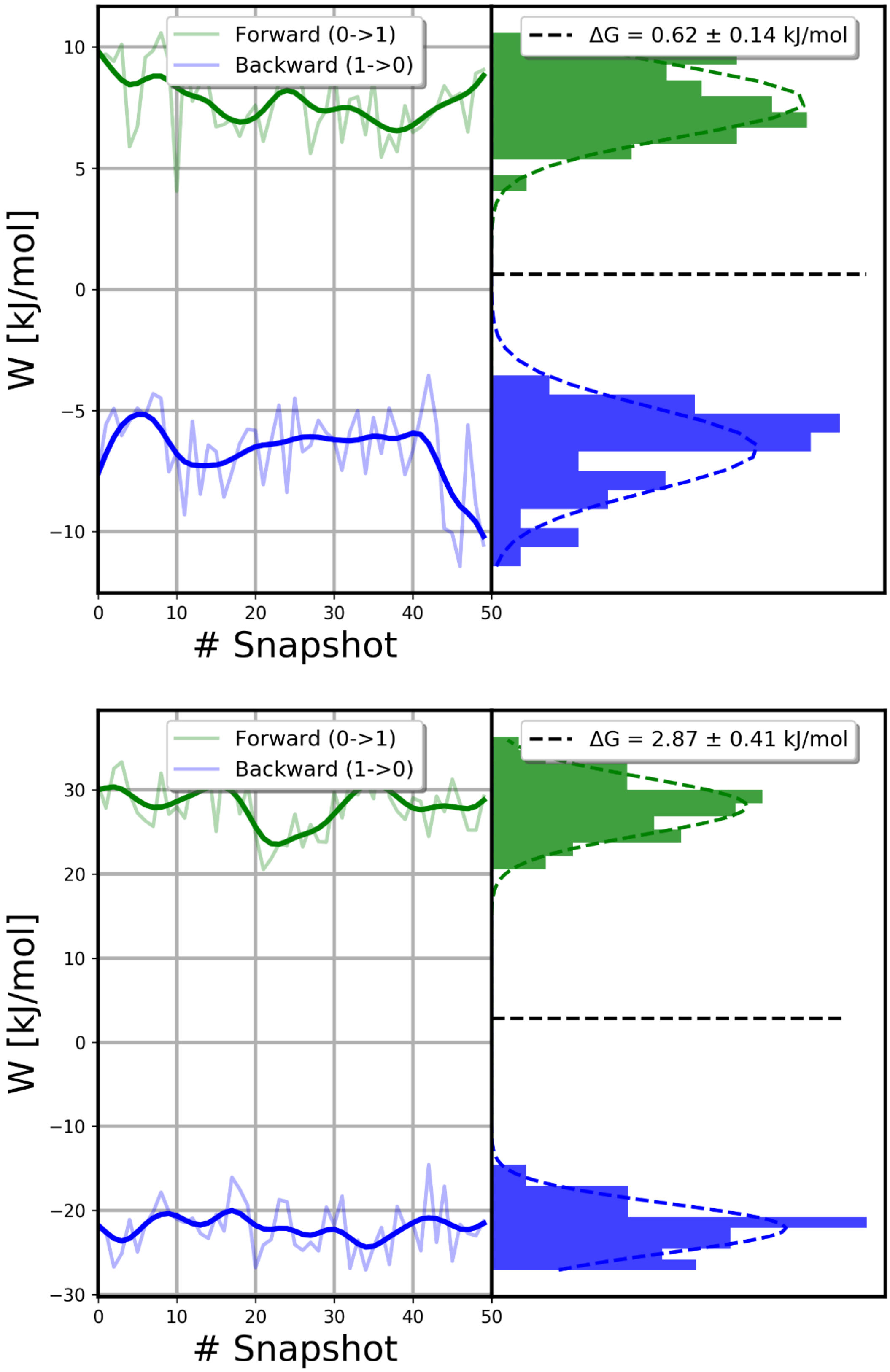


***Figure S12.*** *Free energy plots for unbound (top) and bound (bottom) Y505HID (each plot is the sum of 50 forward and 50 backward 5-ns non-equilibrium MD simulations).* ***Left:*** *Work values for the forward and backward transitions are plotted for every snapshot structure.* ***Right:*** *The work value distributions are depicted as histograms; their intersection gives the free energy difference.*

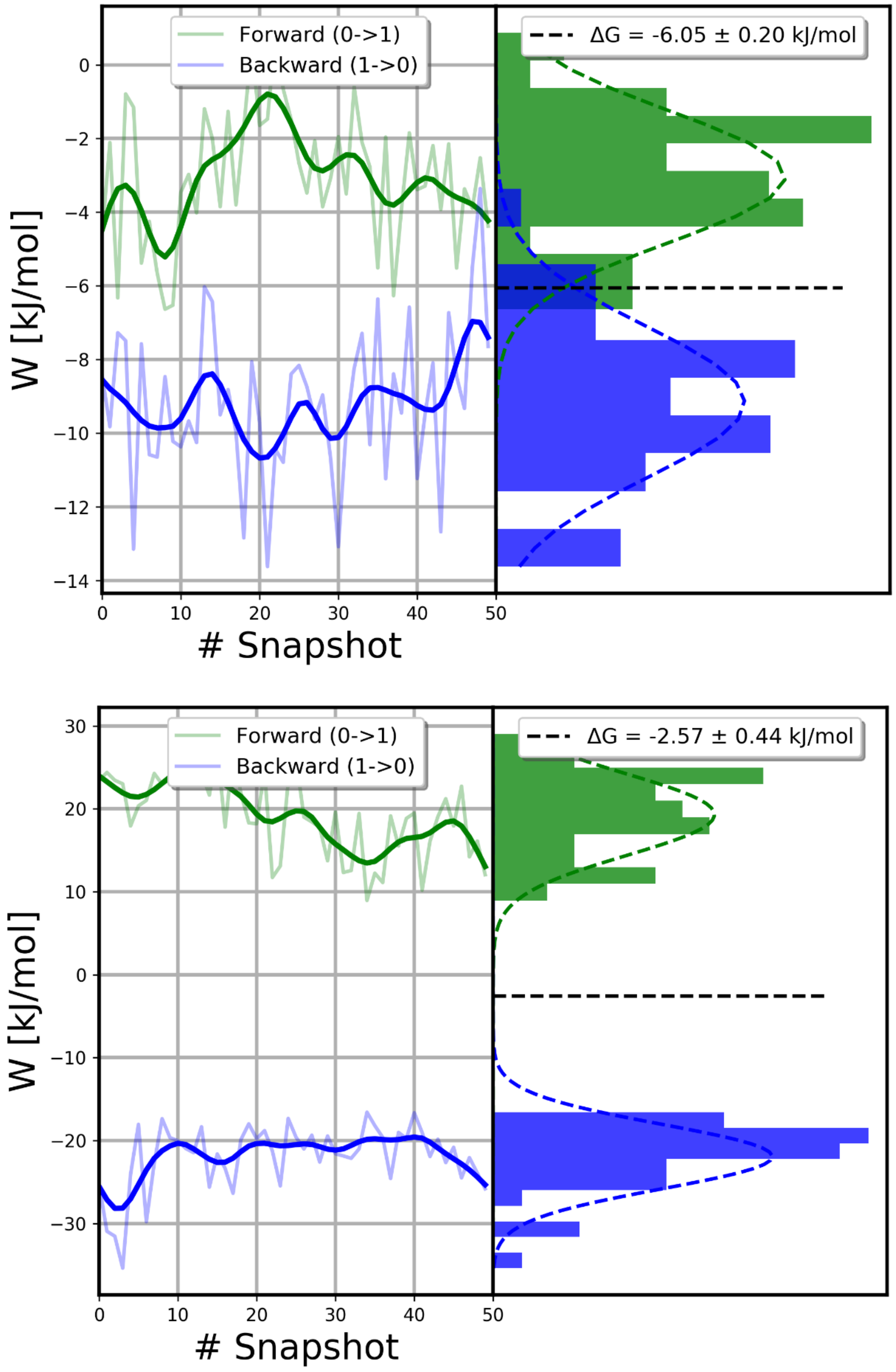


***Figure S13.*** *Free energy plots for unbound (top) and bound (bottom) Y505HIE (each plot is the sum of 50 forward and 50 backward 5-ns non-equilibrium MD simulations).* ***Left:*** *Work values for the forward and backward transitions are plotted for every snapshot structure.* ***Right:*** *The work value distributions are depicted as histograms; their intersection gives the free energy difference.*

**Input scripts for alchemical free energy calculations:**

***A. Forward***

*;=========================================================*
*; Non-equilibrium transition*
*;=========================================================*

*; RUN CONTROL*
*;----------------------------------------------------*
*integrator = sd ; stochastic leap-frog integrator*
*nsteps = 2500000 ; 2 * 2500000 fs = 5 ns*
*dt = 0.002 ; 2 fs*
*comm-mode = Linear ; remove center of mass translation*
*nstcomm = 1 ; frequency for center of mass motion removal*

*; OUTPUT CONTROL*
*;----------------------------------------------------*
*nstxout = 10000 ; save coordinates to .trr every 20 ps*
*nstvout = 10000 ; save velocities to .trr every 20 ps*
*nstfout = 10000 ; save forces to .trr every 20 ps*
*nstxout-compressed = 10000 ; xtc compressed trajectory output every 20 ps*
*compressed-x-precision = 1000 ; precision with which to write to the compressed trajectory file*
*nstlog = 5000 ; update log file every 10 ps*
*nstenergy = 1000 ; save energies every 2 ps*
*nstcalcenergy = 1 ; calculate energies at every step*

*; BONDS*
*;----------------------------------------------------*
*constraint_algorithm = lincs ; holonomic constraints*
*constraints = all-bonds ; constrain all-bonds*
*lincs-iter = 2 ; accuracy of LINCS (1 is default)*
*lincs-order = 6 ; also related to accuracy (4 is default)*
*lincs-warnangle = 30 ; maximum angle that a bond can rotate before LINCS will complain (30 is default)*
*continuation = yes ; formerly known as 'unconstrained-start' - useful for exact continuations and reruns*

*; NEIGHBOR SEARCHING*
*;----------------------------------------------------*
*cutoff-scheme = Verlet ; group or Verlet*
*ns-type = grid ; search neighboring grid cells*
*nstlist = 10 ; 20 fs (default is 10)*
*rlist = 1.0 ; short-range neighborlist cutoff (in nm)*
*pbc = xyz ; 3D PBC*

*; ELECTROSTATICS & EWALD*
*;----------------------------------------------------*
*coulombtype = PME ; Particle Mesh Ewald for long-range electrostatics*

```
coulomb-modifier = Potential-shift-Verlet
rcoulomb         = 1.0                   ; short-range electrostatic cutoff (in nm)
ewald-geometry   = 3d                    ; Ewald sum is performed in all three dimensions
pme-order        = 4                     ; interpolation order for PME (default is 4)
fourierspacing   = 0.12                  ; grid spacing for FFT
ewald-rtol       = 1e-5                  ; relative strength of the Ewald-shifted direct potential at
   rcoulomb

; VAN DER WAALS
;----------------------------------------------------
vdw-type         = Cut-off      ; potential switched off at rvdw-switch to reach zero at rvdw
vdw-modifier     = Potential-shift-Verlet
rvdw             = 1.0          ; van der Waals cutoff (in nm)
DispCorr         = EnerPres     ; apply analytical long range dispersion corrections for
   Energy and Pressure

; TEMPERATURE COUPLING (Langevin)
;----------------------------------------------------
tc-grps          = System
tau-t            = 1.0
ref-t            = 298.15
gen-vel          = no           ; Velocity generation (if gen-vel is 'yes', continuation should be
   'no')
gen-seed         = -1

; PRESSURE COUPLING
;----------------------------------------------------
pcoupl           = Parrinello-Rahman
pcoupltype       = isotropic           ; uniform scaling of box vectors
tau_p            = 2.0                 ; time constant (ps)
ref_p            = 1.0                 ; reference pressure (bar)
compressibility  = 4.5e-05             ; isothermal compressibility of water (bar^-1)
refcoord-scaling = no

; FREE ENERGY
;----------------------------------------------------
free-energy      = yes
init-lambda      = 0     ; start from state A
delta-lambda     = 2.5e-07  ; complete transition in this number of steps
sc-coul          = yes   ; use soft-core also for coulombic interactions
sc-alpha         = 0.3   ; soft-core
sc-sigma         = 0.25  ;
sc-power         = 1     ;
nstdhdl          = 1     ; write to dhdl at each step
```

**B. Reverse**

```
;====================================================
; Non-equilibrium transition
;====================================================
```

```
; RUN CONTROL
;---------------------------------------------------
integrator   = sd           ; stochastic leap-frog integrator
nsteps       = 2500000       ; 2 * 2500000 fs = 5 ns
dt           = 0.002         ; 2 fs
comm-mode    = Linear        ; remove center of mass translation
nstcomm      = 1             ; frequency for center of mass motion removal

; OUTPUT CONTROL
;---------------------------------------------------
nstxout                = 10000     ; save coordinates to .trr every 20 ps
nstvout                = 10000     ; save velocities to .trr every 20 ps
nstfout                = 10000     ; save forces to .trr every 20 ps
nstxout-compressed      = 10000     ; xtc compressed trajectory output every 20 ps
compressed-x-precision  = 1000      ; precision with which to write to the compressed
   trajectory file
nstlog                 = 5000      ; update log file every 10 ps
nstenergy              = 1000      ; save energies every 2 ps
nstcalcenergy          = 1         ; calculate energies at every step

; BONDS
;---------------------------------------------------
constraint_algorithm   = lincs      ; holonomic constraints
constraints           = all-bonds  ; constrain all bonds
lincs-iter            = 2          ; accuracy of LINCS (1 is default)
lincs-order           = 6          ; also related to accuracy (4 is default)
lincs-warnangle        = 30         ; maximum angle that a bond can rotate before LINCS will
   complain (30 is default)
continuation           = yes        ; formerly known as 'unconstrained-start' - useful for exact
   continuations and reruns

; NEIGHBOR SEARCHING
;---------------------------------------------------
cutoff-scheme         = Verlet ; group or Verlet
ns-type              = grid   ; search neighboring grid cells
nstlist              = 10     ; 20 fs (default is 10)
rlist                = 1.0    ; short-range neighborlist cutoff (in nm)
pbc                  = xyz    ; 3D PBC

; ELECTROSTATICS & EWALD
;---------------------------------------------------
coulombtype      = PME                    ; Particle Mesh Ewald for long-range electrostatics
coulomb-modifier = Potential-shift-Verlet
rcoulomb         = 1.0                    ; short-range electrostatic cutoff (in nm)
ewald-geometry   = 3d                     ; Ewald sum is performed in all three dimensions
pme-order        = 4                      ; interpolation order for PME (default is 4)
fourierspacing   = 0.12                   ; grid spacing for FFT
ewald-rtol       = 1e-5                   ; relative strength of the Ewald-shifted direct potential at
   rcoulomb

; VAN DER WAALS
```

*;---------------------------------------------------*
*vdw-type = Cut-off ; potential switched off at rvdw-switch to reach zero at rvdw*
*vdw-modifier = Potential-shift-Verlet*
*rvdw = 1.0 ; van der Waals cutoff (in nm)*
*DispCorr = EnerPres ; apply analytical long range dispersion corrections for Energy and Pressure*

*; TEMPERATURE COUPLING (Langevin)*
*;---------------------------------------------------*
*tc-grps = System*
*tau-t = 1.0*
*ref-t = 298.15*
*gen-vel = no ; Velocity generation (if gen-vel is 'yes', continuation should be 'no')*
*gen-seed = -1*

*; PRESSURE COUPLING*
*;---------------------------------------------------*
*pcoupl = Parrinello-Rahman*
*pcoupltype = isotropic ; uniform scaling of box vectors*
*tau_p = 2.0 ; time constant (ps)*
*ref_p = 1.0 ; reference pressure (bar)*
*compressibility = 4.5e-05 ; isothermal compressibility of water (bar^-1)*
*refcoord-scaling = no*

*; FREE ENERGY*
*;---------------------------------------------------*
*free-energy = yes*
*init-lambda = 1 ; start from state B*
*delta-lambda = -2.5e-07 ; complete transition in this number of steps*
*sc-coul = yes ; use soft-core also for coulombic interactions*
*sc-alpha = 0.3 ; soft-core*
*sc-sigma = 0.25 ;*
*sc-power = 1 ;*
*nstdhdl = 1 ; write to dhdl at each step*
